# Cell-sized asymmetric phospholipid-amphiphilic protein vesicles with growth, fission, and molecule transportation

**DOI:** 10.1016/j.isci.2023.106086

**Published:** 2023-01-31

**Authors:** Masato Suzuki, Koki Kamiya

**Affiliations:** 1 Division of Molecular Science, Graduate School of Science and Technology, Gunma University, 1-5-1 Tenjin-Cho, Kiryu, Gunma 376-8515, Japan

**Keywords:** Biochemistry, Cell biology, Membranes, Synthetic biology

## Abstract

Lipid vesicles, which mimic cell membranes in structure and components, have been used to study the origin of life and artificial cell construction. A different approach to developing cell-mimicking systems focuses on the formation of protein- or polypeptide-based vesicles. However, micro-sized protein vesicles that are similar in membrane dynamics to the cell and that reconstitute membrane proteins are difficult to form. In this study, we generated cell-sized asymmetric phospholipid-amphiphilic protein (oleosin) vesicles that allow the reconstitution of membrane proteins and the growth and fission of vesicles. These vesicles are composed of a lipid membrane on the outer leaflet and an oleosin membrane on the inner leaflet. Further, we elucidated a mechanism for the growth and fission of cell-sized asymmetric phospholipid-oleosin vesicles by feeding phospholipid micelles. Our asymmetric phospholipid-oleosin vesicles with the advantages of the lipid leaflet and the protein leaflet will potentially promote understanding of biochemistry and synthetic biology.

## Highlights

• Asymmetric vesicles have lipid on the outer leaflet and oleosin on the inner leaflet
• Membrane proteins are reconstituted into the asymmetric vesicle membranes
• The asymmetric vesicles were grown and divided by feeding lyso-lipid micelles

## Graphical abstract

**Figure:**
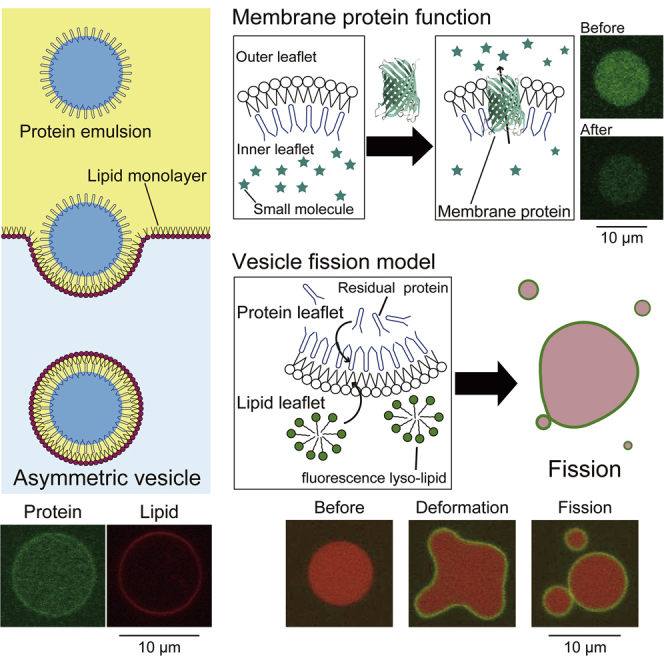

## Introduction

Lipid vesicles, or liposomes, which mimic cell membranes in structure and components, have been widely used to study the origin of life, artificial cell construction, and drug delivery carriers.^1^^,^^2^^,^^3^^,^^4^^,^^5^^,^^6^ The use of lipid vesicles promotes an understanding of essential cellular functions, including membrane protein functions, membrane fusion and fission, complex enzymatic reactions, and protein expression.^7^^,^^8^^,^^9^^,^^10^^,^^11^^,^^12^ Recently, asymmetric lipid vesicles that have different lipid compositions in their outer and inner leaflets and that closely emulate eukaryotic cell membranes have been developed using microfluidic technologies such as pulsed jetting^13^^,^^14^ and droplet transfer methods^15^^,^^16^^,^^17^; consequently, more well-defined complex artificial cell models can be produced.^18^^,^^19^^,^^20^^,^^21^ A different approach to cell-mimicking systems focuses on the formation of protein- or peptide-based vesicles.^22^^,^^23^^,^^24^^,^^25^^,^^26^^,^^27^^,^^28^ Especially, amphiphilic elastin-like polypeptides (ELPs) are assembled into polypeptide vesicles.^29^^,^^30^^,^^31^^,^^32^^,^^33^^,^^34^ New membrane-forming ELPs are generated by cell-free synthesis systems that are enclosed inside ELP vesicles and lead to vesicle growth.^35^ Vesicle growth is a huge advantage of polypeptide vesicles because it does not require any lipid metabolism; only polypeptides are generated by the simple cell-free synthesis reaction as compared with metabolic enzyme reactions. Polypeptide vesicles containing genetic templates for synthesizing polypeptides generate self-replicating protocellular compartments.^36^ However, it is not easy to integrate membrane proteins for transmembrane molecule exchanges, external environment sensing, and energy production into the polypeptide- or protein-vesicles because their physical properties are largely different from those of the lipid vesicles. Therefore, in the future, to create self-replicating protocells, the functional vesicles that enable molecule transportation via the membrane proteins, and the growth and fission of the vesicles, are required for the formation of vesicles with advantages of the lipid and polypeptide- or protein-vesicles.

**Figure 1:**
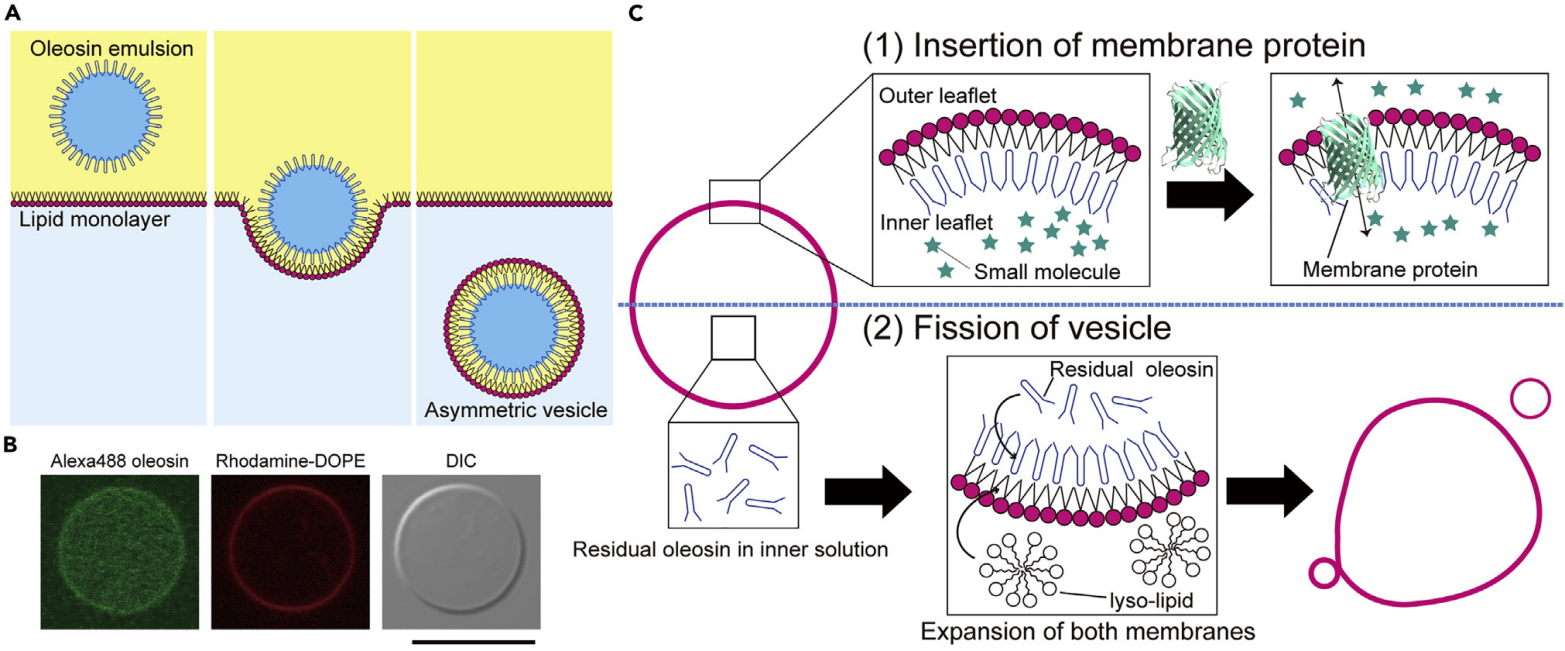
Overview of formation and application of asymmetric lipid-oleosin vesicles (A) Schematic representation of asymmetric lipid-oleosin vesicle formation using the droplet transfer method. (B) Typical confocal images of vesicles composed of lipids and oleosins. DIC represents differential interference contrast. Scale bar, 10 μm. (C) The lipid-oleosin vesicles demonstrated the insertion of membrane proteins (1) and fission of vesicles by the addition of lyso-lipid (2).

In this study, we develop a generation of cell-sized asymmetric phospholipid-amphiphilic protein vesicles that allow the reconstitution of membrane proteins and the growth and fission of vesicles (Figure 1). We use oleosins as amphiphile proteins (Figure S1), which are a type of plant protein whose biological role is to stabilize the oil body.^37^^,^^38^ The formation of cell-sized asymmetric phospholipid-oleosin vesicles composed of phospholipids on the outer leaflet and oleosins on the inner leaflet relies on the droplet transfer method, which is often used for the formation of cell-sized lipid vesicles. Oleosins, which take advantage of amphiphilic characteristics, dissolved in the water phase form water-in-oil (w/o) emulsions with oleosin between the water and oil interface. Cell-sized asymmetric phospholipid-oleosin vesicles are generated by transferring oleosin emulsions from the oil phase to the water phase through the phospholipid monolayers. First, the asymmetricity of the generated cell-sized phospholipid-oleosin vesicles is investigated using a fluorescence quenching assay of NBD-conjugated phospholipids on the outer leaflet. Fundamental properties of the asymmetric lipid-oleosin vesicle membrane toward the complex self-replicating protocells were investigated, including long-term stability, membrane diffusion, membrane thickness, and membrane permeability. Next, the functional membrane proteins are reconstituted into asymmetric phospholipid-oleosin vesicle membranes. Finally, we demonstrate the growth and fission of cell-sized asymmetric phospholipid-oleosin vesicles by feeding phospholipid micelles.

## Results

### Formation of cell-sized lipid-oleosin vesicles

**Figure 2:**
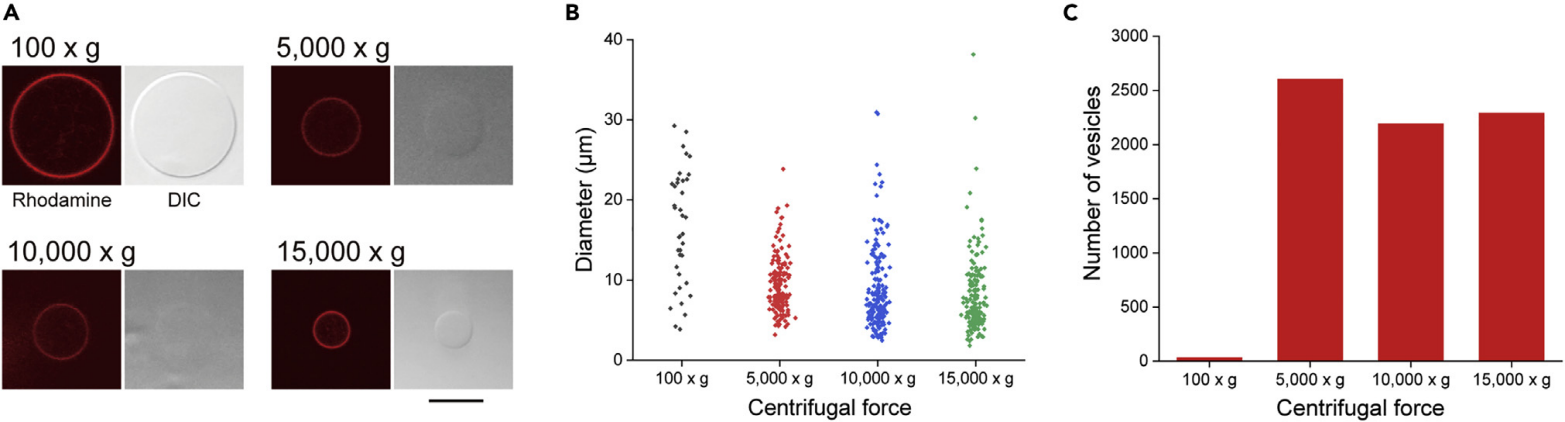
Formation of the lipid-oleosin vesicles at four centrifugal forces (A) Typical confocal images of the lipid-oleosin vesicles prepared by centrifugal forces applied at 100 × *g*, 5,000 × *g*, 10,000 × *g*, and 15,000 × *g* for 10 min. Scale bar, 10 μm. (B) Diameters of the lipid-oleosin vesicles at different centrifugal forces (n = 38 [100 × *g*], 166 [5,000 × *g*], 173 [10,000 × *g*], and 166 [15,000 × *g*]). Three experiments for each parameter. (C) Number of the lipid-oleosin vesicles obtained at each centrifugal force used. Total number of the lipid-oleosin vesicles diluted by a quarter in glucose solution and observed three times. Three experiments for each parameter.

Oleosin, which is used for the formation of cell-sized lipid-oleosin vesicles, was expressed and purified using a method described in a previous study^27^^,^^39^^,^^40^ (Figure S2). The purity of the oleosin protein was confirmed by SDS-PAGE analysis. The band corresponding to purified oleosin protein appeared at approximately 13 kDa (Figure S3). To generate asymmetric lipid-oleosin vesicles composed of lipids on the outer leaflet and oleosin on the inner leaflet, we considered the transfer of oleosin w/o emulsions to the interface of the lipid monolayer. Although giant oleosin vesicles were formed by evaporating the organic solvent from water-in-oil-in-water double emulsions using oleosin dissolved in an organic solvent, oleosin dissolved in phosphate buffer solution was used for SDS-PAGE analysis and CD spectrometry.^41^^,^^42^ As a result of this, oleosin dissolved in an aqueous solution was found to disperse and form a secondary structure. The w/o emulsions were formed from amphiphilic proteins dissolved in a water solution.^28^ In this study, w/o emulsions derived from a mixed solution of mineral oil and oleosin dissolved in HEPES buffer solution containing sucrose (10 mM HEPES, 140 mM NaCl, 500 mM sucrose) were prepared by vortexing. Alexa Fluor 488-conjugated oleosin, which was arranged between the oil-water interface, was observed using a confocal laser-scanning microscope (CLSM; FV-1200, Olympus, Tokyo, Japan) (Figure S4). Oleosin w/o emulsions were also observed. Cell-sized lipid-oleosin vesicles were generated using the droplet -transfer method. The oleosin w/o emulsion consisting of the inner leaflet of the vesicle was added to the oil phase of the 1,2-dioleoyl-*sn*-glycero-3-phosphocholine (DOPC) monolayer consisting of the outer leaflet of the vesicle. After centrifugation, the oleosin emulsion was transferred to the interface of the lipid monolayer between the oil and water phases, and lipid-oleosin vesicles were generated in the water phase (Figure 1A). Rhodamine fluorescence of rhodamine-conjugated 1,2-dioleoyl-*sn*-glycero-3-phosphoethanolamine-N-(lissamine rhodamine B sulfonyl) (Rh-DOPE) and Alexa Fluor 488 fluorescence of oleosins co-localized on the membranes of the formed vesicles (Figure 1B) was observed, suggesting that the vesicle membranes were composed of lipids and oleosins. The size controllability and vesicle productivity of the lipid-oleosin vesicles were investigated by changing the centrifugal force. The average diameters of the lipid-oleosin vesicles formed at 100 × *g*, 5,000 × *g*, 10,000 × *g*, and 15,000 × *g* were 16.6 ± 7.0 μm (mean ± SD), 9.3 ± 3.4 μm (mean ± SD), 8.7 ± 5.1 μm (mean ± SD), and 8.1 ± 4.8 μm (mean ± SD), respectively (Figures 2A and 2B). The diameters of the lipid-oleosin vesicles formed at over 5,000 × *g* were significantly smaller than those formed at 100 × *g*. This result shows that the diameter of the lipid-oleosin vesicles does not change in the range of 5,000 × g to 15,000 × g. Therefore, adjusting the centrifugal force has little effect on controlling the size of the lipid-oleosin vesicles formed by this method. Histograms of the sizes of oleosin emulsions (before centrifugation) and lipid-oleosin vesicles (after centrifugation) are shown in Figure S5. The size distribution of the oleosin emulsions had a wider range (between 2.7 and 67.1 μm in diameter) compared to that of the lipid-oleosin vesicles. The lipid-oleosin vesicles showed a distribution between 4.4 and 41.0 μm in diameter through the droplet transfer process. This change in size distribution between the oleosin emulsions and the lipid-oleosin vesicles corresponds to that of the giant lipid vesicles generated using the droplet transfer method.^43^

We also investigated the relationship between the number of vesicles and the centrifugal force. After centrifugation, 20 μL of the lipid-oleosin vesicle solution was collected at the bottom of the tube and diluted 4-fold with glucose solution. We observed 20 μL of the diluted solution three times and counted the total number of lipid-oleosin vesicles for each parameter. Figure 2C shows the total number of particles produced by the four centrifugal forces. Lipid-oleosin vesicles were generated from 100 × *g* to 15,000 × *g*, with little difference in the number of vesicles generated from 5,000 to 15,000 × *g*. The lipid vesicle formation of the droplet transfer method affected some parameters, such as speed and time of centrifugation, types of organic solvent dissolved in lipids, and density differences between the inside and outside of the vesicles. The formation of the cell-sized lipid vesicles using the droplet transfer method based on mineral oil required 1,600-12,000 × *g* and 10-30 min of centrifugation force and time, respectively.^44^^,^^45^^,^^46^ Under the conditions of cell-sized lipid vesicle formation, a low number of lipid-oleosin vesicles was generated at 100 × *g* due to inefficient transfer of oleosin emulsions across the interface of the lipid monolayer. A wide range of centrifugal speeds was selected to generate lipid-oleosin vesicles depending on the experimental system. Although the distribution of the mean size of lipid-oleosin vesicles was large, in the range of 5,000 × g to 15,000 × g, lipid-oleosin vesicles were generated at 5,200 × *g* in subsequent experiments to produce a sufficient number of lipid-oleosin vesicles to investigate the physical properties of the vesicles.

### Confirmation of asymmetry and membrane diffusion of lipid-oleosin vesicles

**Figure 3:**
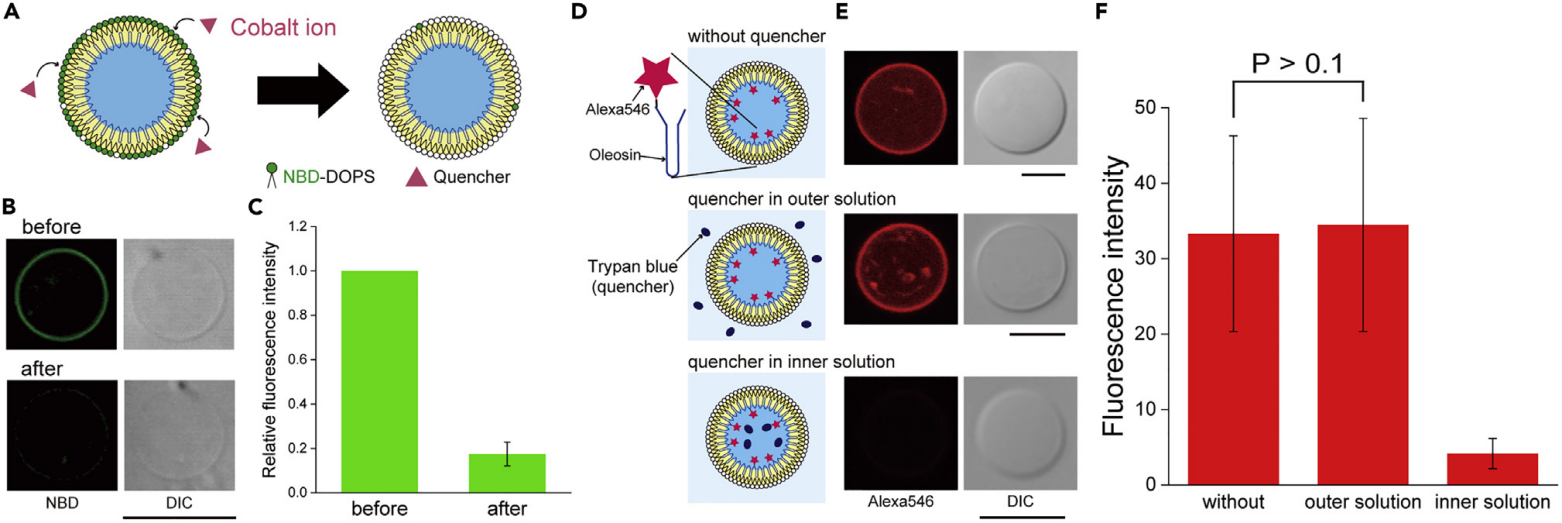
Quenching assay to confirm the asymmetry of the lipid-oleosin vesicles (A) Schematic representation of quenching assay using Cobalt ion (NBD quencher). (B) Typical confocal images of the lipid-oleosin vesicles containing NBD-DOPS on the outer leaflet before and 20 min after the addition of the quencher. Scale bar, 10 μm. (C) Relative fluorescence intensities of NBD of the lipid-oleosin vesicles before (n = 12) and after (n = 12) addition of quencher. Background fluorescence intensities were excluded. Data represented as mean ± SD. (D) Schematic representation of quenching assay using trypan blue (Alexa Fluor 546 quencher). (E) Typical confocal images of the lipid-oleosin vesicles containing Alexa Fluor 546-conjugated oleosin without quencher and with quencher in outer or inner solution. Scale bar, 5 μm. (F) Fluorescence intensities of Alexa Fluor 546-conjugated oleosin on the lipid-oleosin vesicle membranes without quencher (n = 19) and with quencher in outer (n = 15) or inner solution (n = 18). Background fluorescence intensities were excluded. Student’s *t* test. Data represented as mean ± SD.

To confirm the asymmetric distribution of lipids on the outer leaflet of the lipid-oleosin vesicle membranes, a fluorescence quenching assay was performed. Cobalt ion (NBD quencher), a hydrophilic substance that cannot pass through hydrophobic membranes, was added to the outer solution of the lipid-oleosin vesicles (Figure 3A). The use of cobalt ions to determine the asymmetry of 1,2-dioleoyl-*sn*-glycero-3-phospho-L-serine-N-(7-nitro-2-1,3-benzoxadiazol-4-yl) (NBD-DOPS) on the outer leaflet of asymmetric lipid vesicles has been previously reported using this quenching assay.^47^ The NBD fluorescence on the lipid-oleosin vesicle membranes significantly decreased after the addition of cobalt ions (Figure 3B). The relative fluorescence intensity of NBD obtained from the lipid-oleosin vesicles after the addition of cobalt ion was 0.17 ± 0.05 (mean ± SD) (Figure 3C), indicating that approximately 83% of the fluorescence was quenched. When the same concentration of cobalt ions as the NBD quenching assay was added to the outer solution of the cell-sized lipid vesicles containing NBD-DOPS on both leaflets, the relative fluorescence intensity of NBD on the lipid vesicles was 0.58 ± 0.03 (mean ± SD) (Figure S6). This suggests that this quenching assay can confirm whether lipid-oleosin vesicles are asymmetric. Moreover, other quenchers have been used to evaluate the asymmetric distribution of lipids in the outer leaflet of lipid-oleosin vesicle membranes. The use of 2,4,6-trinitrobenzenesulfonic acid sodium (rhodamine quencher) to determine the asymmetry of 1,2-dioleoyl-*sn*-glycero-3-phosphoethanolamine-N-(lissamine rhodamine B sulfonyl) (Rh-DOPE) on the outer leaflet of asymmetric lipid vesicles was reported using this quenching assay.^48^ When rhodamine quencher was added to the outer solution of the lipid-oleosin vesicles containing Rh-DOPE on the outer leaflet, its relative fluorescence intensity after the addition of the rhodamine quencher was 0.21 ± 0.08 (mean ± SD) (Figure S7), indicating that approximately 79% of the fluorescence was quenched. A previous study showed that the quenching rates of asymmetric lipid vesicles containing Rh-DOPE on the outer leaflet were 85%^43^ and 89%,^49^ respectively.

The presence of oleosin proteins in the inner leaflet was also confirmed using a fluorescence quenching assay. Alexa Fluor was quenched by trypan blue.^50^ Trypan blue (Alexa Fluor 546 quencher) was added to the inner or outer solutions of the lipid-oleosin vesicles containing Alexa Fluor 546-conjugated oleosin (Figure 3D). When the quencher was added to the outer solution of the lipid-oleosin vesicles containing Alexa Fluor 546-conjugated oleosin, red fluorescence was observed on the vesicle membranes (Figure 3E). In contrast, when the quencher was added to the inner solution of the lipid-oleosin vesicles containing Alexa Fluor 546-conjugated oleosin, the intensity of red fluorescence was significantly decreased (Figure 3E). The fluorescence intensities of Alexa Fluor 546-conjugated oleosin on the lipid-oleosin vesicle membranes without the quencher and with the quencher in the outer or inner solutions were 33.3 ± 13.0 (mean ± SD), 34.5 ± 14.1 (mean ± SD), and 4.2 ± 2.0 (mean ± SD), respectively (Figure 3F). The fluorescence intensities of Alexa Fluor 546-conjugated oleosin on the lipid-oleosin vesicle membranes with the quencher in the outer solution were not significantly different from those without the quencher. However, the fluorescence intensities of Alexa Fluor 546-conjugated oleosin on the lipid-oleosin vesicle membranes with the quencher in the inner solution decreased to 12% of those without the quencher. These results suggest that almost all of the oleosin proteins exist in the inner leaflet of the lipid-oleosin vesicles. The results obtained in this study indicate that the lipid-oleosin vesicles formed through the droplet transfer method have an asymmetric distribution, containing the lipid on the outer leaflet and oleosin on the inner leaflet.

**Figure 4:**
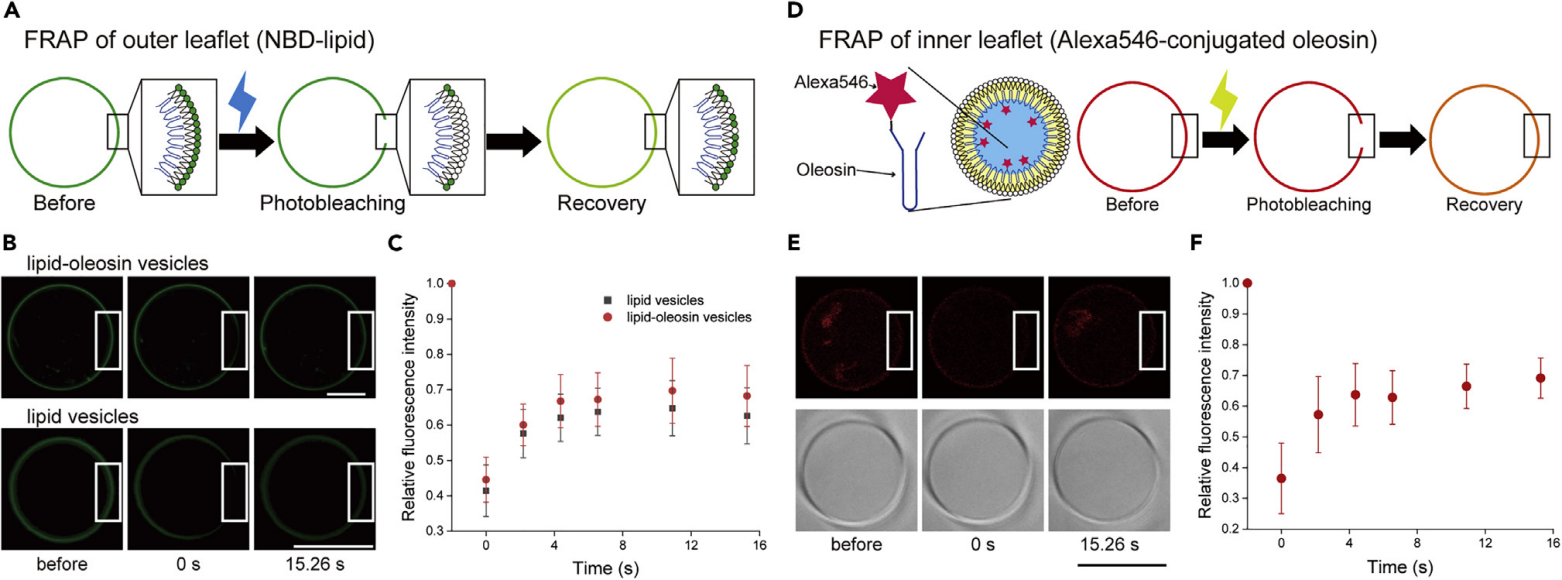
FRAP assay to confirm the membrane diffusion of the lipid-oleosin vesicles (A) Schematic representation of fluorescence recovery after photo bleaching (FRAP) assay of the NBD-lipid on the outer leaflet of the asymmetric lipid-oleosin vesicles. (B) Typical confocal laser scanning microscopy time-lapse images of the asymmetric lipid-oleosin vesicles and lipid vesicles before and after photobleaching. Scale bar, 10 μm. (C) Relative fluorescence intensities of the NBD of the asymmetric lipid-oleosin vesicles (n = 15) and lipid vesicles (n = 12) after photobleaching. Data represented as mean ± SD. (D) Schematic representation of FRAP assay of the Alexa Fluor 546-conjugated oleosin on the inner membranes of the asymmetric lipid-oleosin vesicles. (E) Typical CLSM time-lapse images of the asymmetric lipid-oleosin vesicles containing the Alexa Fluor 546-conjugated oleosin before and after photobleaching. Scale bar, 10 μm. (F) Relative fluorescence intensities of the Alexa Fluor 546-conjugated oleosin on the asymmetric lipid-oleosin vesicle membranes (n = 12) after photobleaching. Data represented as mean ± SD.

We evaluated the mobility of lipids or oleosin in asymmetric lipid-oleosin vesicles using fluorescence recovery after photo bleaching (FRAP). To observe this, a section of NBD-DOPS on the outer leaflet of the asymmetric lipid-oleosin vesicles was quenched using CLSM (Figure 4A). The recovery of NBD fluorescence around the bleached area of the asymmetric lipid-oleosin vesicles was observed (Figures 4B and 4C). The fluorescence recovery was similar to that of the lipid vesicles, suggesting that NBD-DOPS in the asymmetric lipid-oleosin vesicles has lateral diffusion at the same level as NBD-DOPS in the lipid vesicles. The oleosin fluidity on the inner leaflet of the asymmetric lipid-oleosin vesicles was also investigated by FRAP (Figure 4D), and recovery of Alexa Fluor 546 fluorescence around the bleaching area was observed (Figures 4E and 4F). We found that oleosin in the inner leaflet of asymmetric lipid-oleosin vesicles also showed lateral diffusion. Upon comparison of the slopes of the fluorescence recovery graphs of lipids and oleosin in the asymmetric lipid-oleosin vesicles, it was found that the slope of the oleosin graph was in the same range as that of the lipid graph (Figures 4E and 4F).

The membrane stability of the asymmetric lipid-oleosin vesicles was investigated by changing the glucose solution in the outer solution (Figure S8). A high concentration of glucose buffer solution was added to the outer solution of the asymmetric lipid-oleosin vesicles or the lipid vesicles containing 500 mM sucrose buffer solution (inner solution) and 500 mM glucose buffer solution (outer solution) owing to the generation of the osmotic pressure difference. After the addition of a high-concentration glucose buffer solution for 10 min, the vesicles were observed by CLSM. When the glucose concentration of the outer solution increased, the numbers of the asymmetric lipid-oleosin vesicles and lipid vesicles decreased (Figures S8C and S8F). In particular, the number of lipid vesicles in the outer solution at a glucose concentration of 2 M (final concentration) was low (Figure S8F). These results indicate that the membrane stability of asymmetric lipid-oleosin vesicles may be higher than that of lipid vesicles.

### Retention of size and inner solution of asymmetric lipid-oleosin vesicles

**Figure 5:**
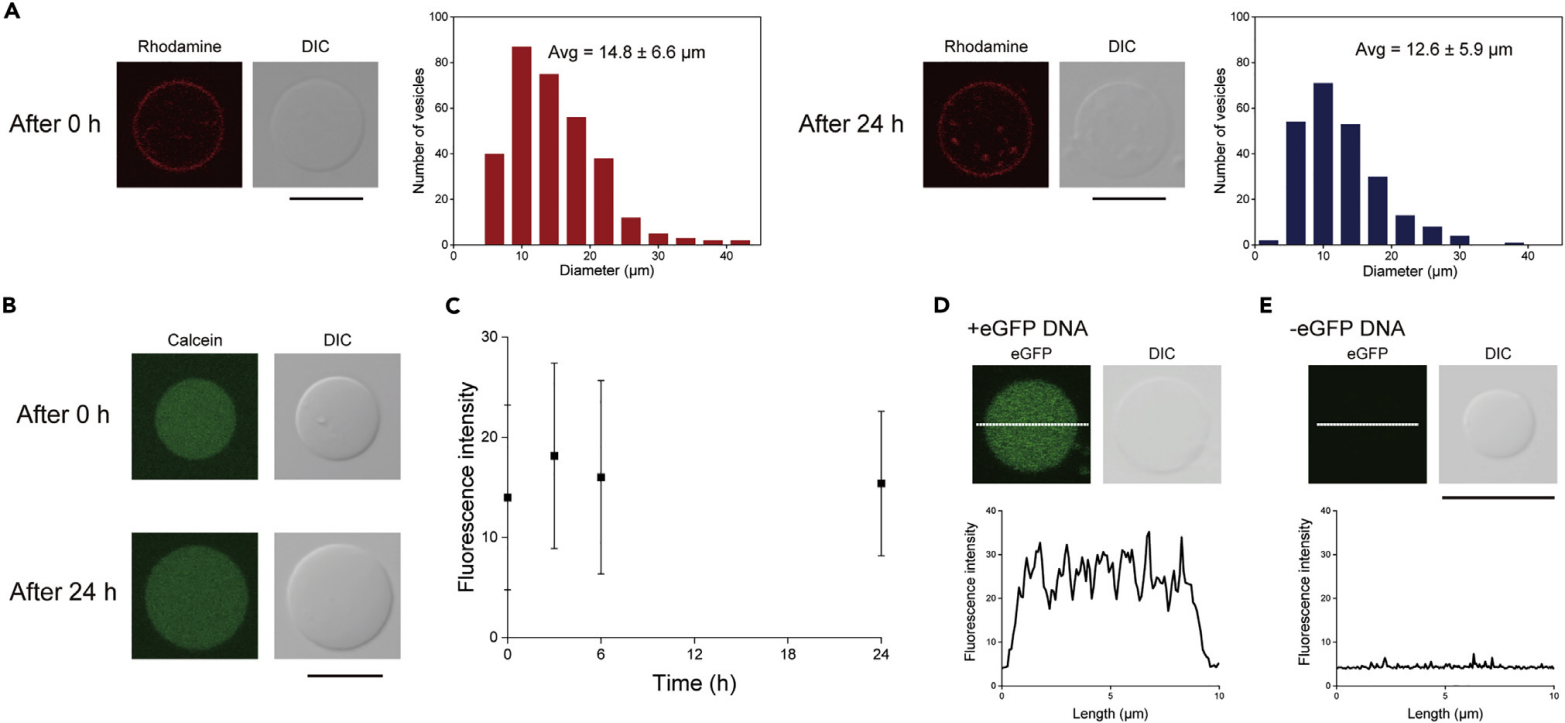
Basic properties of the lipid-oleosin vesicles (A) Typical confocal images and size distribution of the lipid-oleosin vesicles before (n = 320) and after (n = 236) incubation at 37°C for 24 h (new vesicles were observed at each time point). Scale bar, 10 μm. (B) Typical confocal images of the lipid-oleosin vesicles containing calcein before and after incubation at 37°C for 24 h. Scale bar, 10 μm. (C) Average fluorescence intensity values of calcein in the lipid-oleosin vesicles before (n = 66) and after incubation at 37°C for 3 h (n = 66), 6 h (n = 30), and 24 h (n = 67) (new vesicles were observed at each time point). Background fluorescence intensities were excluded. Data represented as mean ± SD. (D and E) Typical confocal images of the asymmetric lipid-oleosin vesicles containing a cell-free synthesis system, with or without enhanced green fluorescent protein-encoded DNA.

Vesicle stability is an important property of enzymatic reactions in the inner phase of the vesicles. We confirmed the retention of the size and inner solution of asymmetric lipid-oleosin vesicles. The diameters of the asymmetric lipid-oleosin vesicles were measured before and after incubation for 24 h at 37°C. The average diameters of the asymmetric lipid-oleosin vesicles before and after incubation were 14.8 ± 6.6 μm (mean ± SD) and 12.6 ± 5.9 μm (mean ± SD), respectively (Figure 5A). The size of the asymmetric lipid-oleosin vesicles was maintained after incubation for 24 h at 37°C. The average diameters of the lipid-oleosin vesicles after incubation for 1 h and 6 h were 15.4 ± 7.0 μm (mean ± SD) and 12.2 ± 5.0 μm (mean ± SD), respectively (Figure S9). At 6 and 24 h, no significant difference in diameter was observed, suggesting that the asymmetric lipid-oleosin vesicles did not shrink. The diameter of the giant lipid vesicles formed by the microfluidic device did not change after incubation for 24 h at 37°C.^13^ Seventy percent of the giant lipid vesicles formed by the droplet transfer method maintained their spherical shape after incubation for 24 h at 37°C.^51^ Therefore, giant asymmetric lipid-oleosin vesicles with a lipid bilayer maintain their stability after formation within 24 h. Moreover, the diameter and the number of the asymmetric lipid-oleosin vesicles after incubation for 7 days at 37°C were 6.9 ± 3.0 μm (mean ± SD) and 61 vesicles, respectively (Figure S10). After incubation for 7 days at 37°C, the vesicle diameter and number decreased because the asymmetric lipid-oleosin vesicles, similar to the lipid vesicles, may rupture or experience fission under longer incubation periods.

Next, the maintenance of the inner solution in the asymmetric lipid-oleosin vesicles was observed by calcein fluorescence leakage. Asymmetric lipid-oleosin vesicles containing calcein were incubated at 37°C for 0, 3, 6, and 24 h. The average fluorescence intensities of calcein obtained from the vesicles were 14.0 ± 9.2 (mean ± SD), 18.1 ± 9.3, 16.0 ± 9.7 (mean ± SD), and 15.4 ± 7.2 (mean ± SD), respectively (Figures 5B and 5C). The fluorescence of calcein in the vesicles incubated for 0 h did not significantly differ from that of vesicles incubated for 24 h. No leakage of small molecules into the asymmetric lipid-oleosin vesicles was observed, suggesting that they were sufficiently robust to withstand general enzymatic reactions.

A cell-free synthesis system was incorporated into the inner solution of asymmetric lipid-oleosin vesicles. The fluorescence of enhanced green fluorescent protein (eGFP) in asymmetric lipid-oleosin vesicles with eGFP-encoded DNA was observed (Figures 5D, 5E, and S11). Although residual oleosin was present in the inner solution, eGFP was synthesized into asymmetric lipid-oleosin vesicles containing the cell-free synthesis system.

### Reconstitution and function of membrane proteins in asymmetric lipid-oleosin vesicles

**Figure 6:**
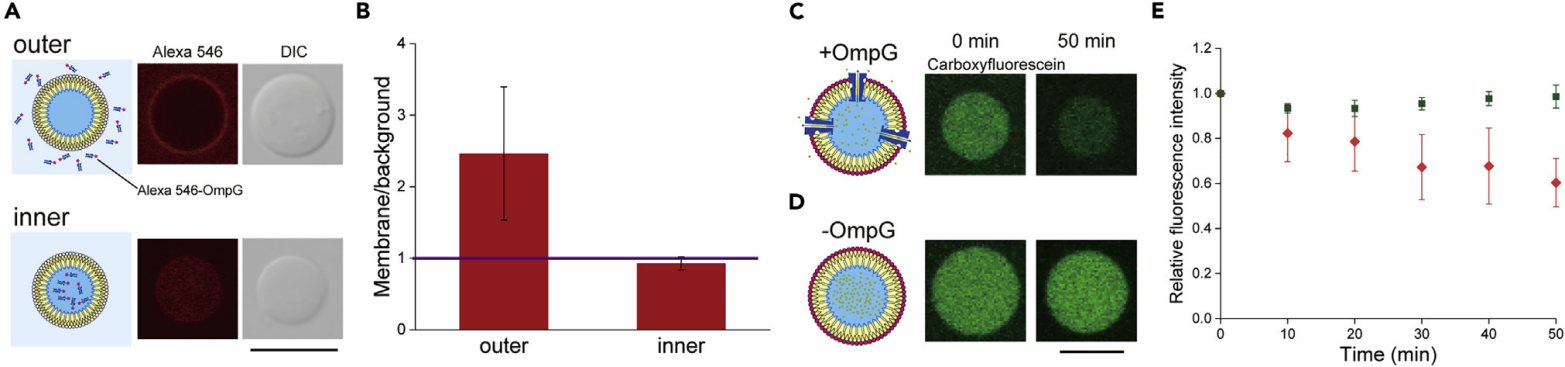
Investigation of insertion and function of outer membrane protein G (OmpG) into lipid-oleosin vesicles (A) Alexa Fluor 546-conjugated OmpG was added to the outer solution or inner solution of lipid-oleosin vesicles. Typical confocal images of the lipid-oleosin vesicles with Alexa Fluor 546-conjugated OmpG from the outside and inside. Scale bar, 10 μm. (B) Membrane fluorescence intensity of the lipid-oleosin vesicles with Alexa Fluor 546-conjugated OmpG from the outside (n = 50) and inside (n = 49) was normalized by the background fluorescence intensity. The purple line represents the value of 1 of the normalized fluorescence intensity. Data represented as mean ± SD. (C and D) Typical confocal laser scanning microscopy fluorescence images of carboxyfluorescein in the lipid-oleosin vesicles with or without OmpG. Scale bar, 10 μm. (E) Relative fluorescence intensities of carboxyfluorescein in the lipid-oleosin vesicles with OmpG (orange diamonds; n = 16 [0 min], 13 [10, 20 min], 11 [30 min], 6 [40 min], 5 [50 min]) or without OmpG (green squares; n = 21 [0, 10 min], 18 [20 min], 17 [30-50 min]). The fluorescence intensity at each point was normalized by the 0 min value. Data represented as mean ± SD.

To examine the insertion of membrane proteins into asymmetric lipid-oleosin membranes, Alexa Fluor 546-conjugated outer membrane protein G (OmpG) was added to the inner or outer solution of the asymmetric lipid-oleosin vesicles. To evaluate OmpG insertion, the fluorescence intensity of OmpG on the vesicle membranes was normalized to the background fluorescence intensity. More than one of the normalized fluorescence intensities showed that OmpG was reconstituted into asymmetric lipid-oleosin membranes. When Alexa Fluor 546-conjugated OmpG was added to the outer solution of asymmetric lipid-oleosin vesicles, red fluorescence of OmpG on the vesicle membranes was observed (Figure 6A), in contrast to its addition to the inner solution. OmpG was inserted only from the side of the lipid leaflets of the asymmetric lipid-oleosin vesicles (Figure 6B), suggesting that the insertion of membrane proteins into the vesicles was advantageous for the lipid leaflet on the outer leaflet. The amount of OmpG inserted into the asymmetric lipid-oleosin vesicles was slightly larger than that inserted into the giant lipid vesicles (Figure S12). To insert membrane proteins into vesicles, the lipid leaflet in the outer leaflet may play a role in the initial stage of membrane protein insertion, such as lipid-membrane protein interactions, and the membrane fluidity of the oleosin leaflet in the inner leaflet may also accelerate the insertion of membrane proteins.

OmpG forms nanopores in the cell membrane and transports ions and small molecules.^52^ The function of OmpG on asymmetric lipid-oleosin vesicles was observed by the transportation of carboxyfluorescein from the inner phase to the outer phase of the vesicles. After incubation for 50 min, the relative fluorescence intensities of carboxyfluorescein in the asymmetric lipid-oleosin vesicles with and without OmpG were 0.60 ± 0.11 (mean ± SD) and 0.99 ± 0.05 (mean ± SD), respectively (Figures 6C-6E), indicating that the function of membrane proteins can be observed on asymmetric lipid-oleosin vesicles. The fluorescence intensity of carboxyfluorescein in the oleosin vesicles with n-octyl-β-D-glucoside (OG) in the outer solution did not change (Figure S13). At least these asymmetric lipid-oleosin vesicles, which undergo small molecule transportation via OmpG, are unilamellar and may have a membrane thickness close to that of cell membranes. The lipid-oleosin membrane may accumulate membrane proteins and become a reaction field for membrane proteins.

### Growth and fission of asymmetric lipid-oleosin vesicles after the addition of lysophosphatidylcholine micelles

**Figure 7:**
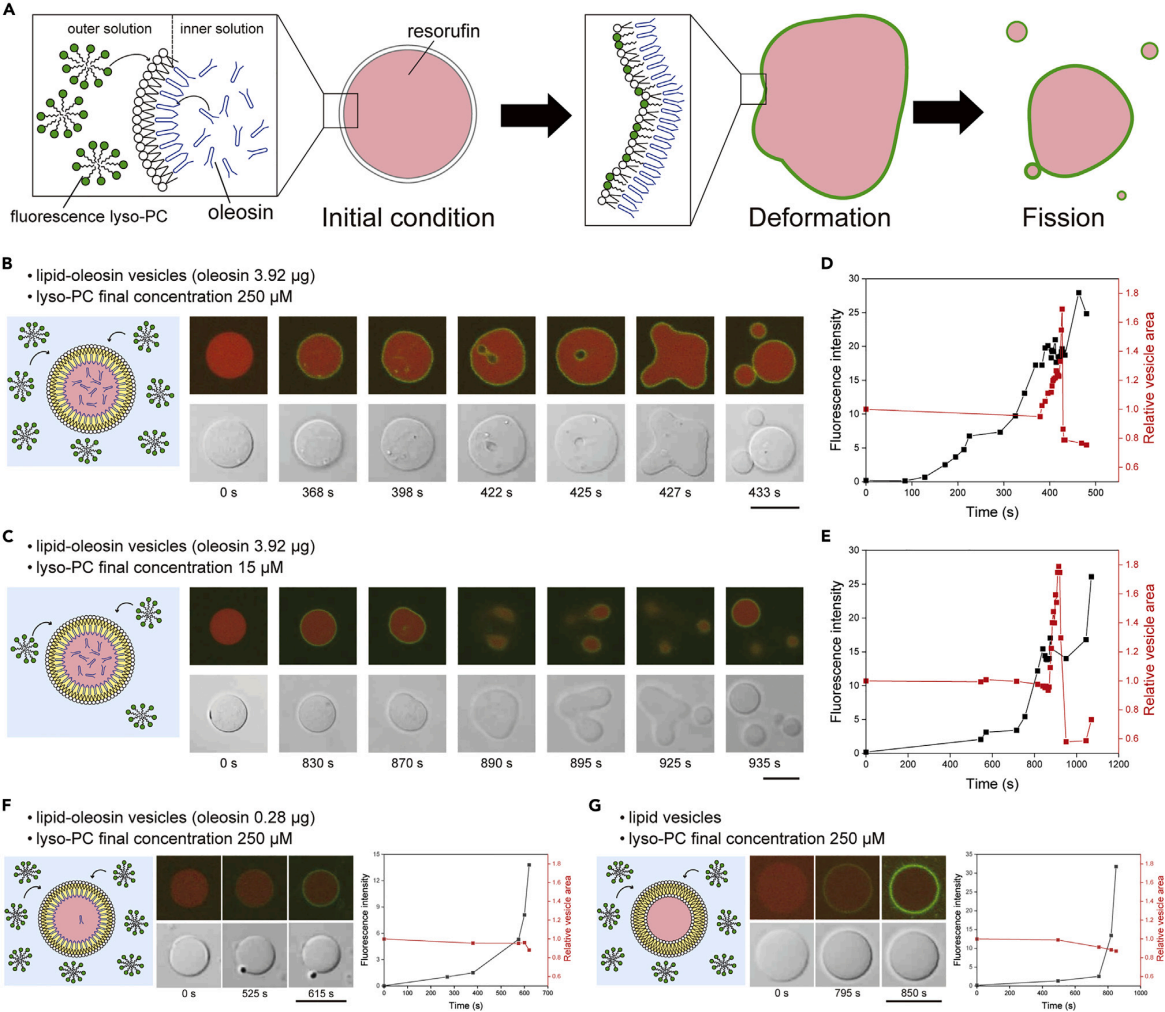
Deformation and fission of lipid-oleosin vesicles containing lysophosphatidylcholine (lyso-PC) (A) Schematic representation of reaction after adding TopFluor lyso-PC micelles to the lipid-oleosin vesicle containing resorufin. (B and C) Typical confocal laser scanning microscopy time-lapse images of deformation and fission of a lipid-oleosin vesicle after the addition of TopFluor lyso-PC micelles (final concentration: 250 μM and 15 μM). Scale bar, 10 μm. (D and E) Fluorescence intensities of the TopFluor lyso-PC on the lipid-oleosin vesicle membranes and relative vesicle area of the lipid-oleosin vesicles after the addition of TopFluor lyso-PC (final lyso-PC concentration: 250 μM and 15 μM). (F) Typical CLSM time-lapse images, relative vesicle area, and fluorescence intensities of the TopFluor lyso-PC on the lipid-oleosin vesicle membrane containing 0.28 μg oleosin after the addition of TopFluor lyso-PC micelles (final concentration: 250 μM). (G) Typical CLSM time-lapse images, relative vesicle area, and fluorescence intensities of the TopFluor lyso-PC on the lipid vesicle membrane after the addition of TopFluor lyso-PC micelles (final concentration: 250 μM).

The realization of artificial cell systems for the growth and fission of giant lipid vesicles is more complex when encapsulating lipid metabolic reactions and fission-related proteins in lipid vesicles. Here, we demonstrate a simple growth and fission system using asymmetric lipid-oleosin vesicles by the addition of lysophosphatidylcholine (lyso-PC) micelles to the outer solution of asymmetric vesicles (Figure 7A).

When lyso-PC micelles containing TopFluor lyso-PC (final concentration 250 μM) were added to the outer solution of the asymmetric lipid-oleosin vesicles containing resorufin, the green fluorescence of TopFluor on the asymmetric lipid-oleosin vesicle membranes was observed after addition for approximately 150 s. After that, the size of the asymmetric lipid-oleosin vesicles increased with an increase in TopFluor fluorescence. The fluorescence intensity of resorufin in the vesicles decreased, suggesting that the size of the vesicles had increased (Table S1 and Figure S14). Eventually, several small vesicles were generated through the deformation of the asymmetric lipid-oleosin vesicles (three experiments) (Figure 7B, Video S1, and Figure S15). After deformation and fission, the asymmetric lipid-oleosin vesicles became spherical. Red fluorescence of resorufin was detected in the inner solution of divided small vesicles. The inner solution was also transferred from the asymmetric lipid-oleosin vesicles to the small vesicles. This phenomenon of growth and fission was caused by the addition of micelles without TopFluor lyso-PC into the asymmetric lipid-oleosin vesicle without resorufin (Figure S16). This growth and fission of the vesicles were not due to the fluorescence probes. When a low concentration (final concentration 15 μM) of micelles was added to the outer solution of the asymmetric vesicles, small vesicles were also generated (four experiments) (Figure 7C, Video S2, and Figure S17).

Video S1. Video of deformation and fission of lipid-oleosin vesicles containing 3.92 μg oleosin and 250 μM lyso-PC (final concentration), related to Figure 7

Video S2. Video of deformation and fission of lipid-oleosin vesicles containing 3.92 μg oleosin and 15 μM lyso-PC (final concentration), related to Figure 7

When the asymmetric lipid-oleosin vesicle membrane reached a fluorescence intensity greater than 10.7 (lyso-PC concentration of the vesicle membrane was 2.67 μM, estimated by a calibration curve, Figure S18), there was a dynamic change in morphology of the asymmetric lipid-oleosin vesicles at both concentrations of micelles (Figures 7D and 7E). The time of maximal growth of vesicles was approximately 400 s and 850 s at final micelle concentrations of 250 μM and 15 μM, respectively (Figures 7D and 7E). The time of maximal growth depends on the concentration of the micelles. When the maximal relative vesicle area was greater than 1.69, some large vesicles (2.5 μm in diameter) were generated from the asymmetric lipid-oleosin vesicle owing to strong deformation (Tables S2 and S3).

However, for the asymmetric lipid-oleosin vesicles containing a low concentration of oleosin (0.28 μg) (the minimum concentration for generating oleosin in w/o emulsion), despite reaching a fluorescence intensity on the asymmetric lipid-oleosin vesicle membrane greater than 12, there was no increase in the size or deformation for asymmetric lipid-oleosin vesicles (nine experiments) (Figure 7F, Video S3, and Figure S19). The lipid vesicles were also not deformed by the addition of micelles, although green fluorescence of TopFluor on the lipid vesicle membranes was observed (six experiments) (Figure 7G, Video S4, and Figure S20). These results suggest that the activation of a simple growth and fission system requires micelles in the outer solution and oleosin in the inner solution of the asymmetric lipid-oleosin vesicle. Based on the results of these experiments, the growth mechanism of the vesicles may be as follows: When lyso-PC micelles are fed to the outer solution of the asymmetric vesicles, the insertion of lyso-PC into the lipid leaflet on the outer leaflet induces an increase in the lipid leaflet area. By increasing the lipid leaflet area, the insertion of residual oleosin into the oleosin leaflet on the inner leaflet may increase the oleosin leaflet area at the same time. This growth mechanism takes advantage of the lipid leaflet on the outer leaflet and the oleosin leaflet on the inner leaflet of asymmetric lipid-oleosin vesicles. After the growth of these vesicles, an increase in lipid leaflet instability leads to vesicle deformation and fission. Although vesicle growth was demonstrated by polypeptide vesicles, vesicle growth, and fission were first achieved by asymmetric lipid-oleosin vesicles that utilized micelles and residual amphiphilic proteins. This experiment showed that asymmetric lipid-oleosin vesicles can be used as models for cell growth and fission.

Video S3. Video of deformation and fission of lipid-oleosin vesicles containing 0.28 μg oleosin and 250 μM lyso-PC (final concentration), related to Figure 7

Video S4. Video of deformation and fission of liposomes containing 250 μM lyso-PC (final concentration), related to Figure 7

## Discussion

In this study, we report the formation of a cell-sized asymmetric lipid-oleosin vesicle composed of a lipid leaflet on the outer leaflet and an oleosin leaflet on the inner leaflet. The fundamental properties of the asymmetric lipid-oleosin vesicles, such as the size distribution and stability of the vesicles, and membrane permeability, thickness, fluidity, and stability, were investigated. These properties allow for the application of biological or chemical reactions and emulations of cell functions, such as the reconstitution and fission of membrane proteins and the growth and fission of vesicles. Of the nanopore proteins that were reconstituted into the asymmetric lipid-oleosin vesicle membrane, OmpG functioned as a small-molecule transport protein. Finally, a simple cell growth and fission model using the asymmetric lipid-oleosin vesicle was demonstrated using only lipids and oleosin, an amphiphilic protein.

To understand the role of oleosin in the promotion of asymmetric lipid-oleosin vesicle fission, we measured the fluorescence intensities of the membrane and inner solution of the oleosin emulsions and the diameters of the oleosin emulsions by changing the oleosin concentration. When the oleosin concentration increased, the fluorescence intensity of the inner solution of the oleosin emulsions increased. In contrast, the fluorescence intensity of the membranes and the diameter of the oleosin emulsions did not change (Figure S21). It was reported that the diameter of protein emulsions did not change when the protein concentration increased.^53^ The constant fluorescence intensity of the membranes indicates the saturation of the oleosin concentration in the membrane. Therefore, the amount of residual oleosin in the inner solution increases with increasing oleosin concentration. We found that the growth and fission of asymmetric vesicles were caused by the amount of residual oleosin in the inner solution. When the increase in the surface area of the lipid membrane on the outer leaflet by the addition of lyso-PC micelles simultaneously causes a sparsity oleosin membrane on the inner leaflet, the oleosin in the inner solution of the asymmetric vesicles may be inserted into the sparse oleosin membranes (Figure S22). FRAP experiments of the asymmetric lipid-oleosin vesicles showed that the lateral fluidity of the lipid in the outer leaflet and oleosin in the inner leaflet also contributed to the growth, deformation, and fission of the asymmetric vesicles. After the growth of these vesicles, an increase in lipid leaflet instability leads to vesicle deformation and fission. The membrane fluidity of oleosin in the inner leaflet also plays an important role in the growth and fission of asymmetric lipid-oleosin vesicles. In addition, we evaluated the structure of oleosin stored in a buffer solution using CD spectroscopy. The CD spectrum of oleosin did not change after purification for approximately 7 days (Figure S23). Although oleosin is an amphiphilic protein, its structure is maintained in the buffer solution. When asymmetric lipid-oleosin vesicles were generated using oleosin stored in a buffer solution for more than 2 weeks, they were hardly deformed by the addition of lyso-PC micelles. The secondary structure of oleosin may be important for the deformation and fission of asymmetric lipid-oleosin vesicles. Therefore, we conclude that residual oleosin can exist in the inner solution of oleosin emulsions.

In conclusion, these asymmetric vesicles have properties of both lipid (e.g., reconstitution/function of membrane proteins and deformation of the membrane) and polypeptide- or protein-vesicles (e.g., intrusion of amphiphilic proteins into the protein leaflets and conjugation of enzymes to amphiphilic proteins). We believe that the asymmetric lipid-oleosin vesicle has the potential as a novel platform for a complex cellular model that combines the advantages of lipid and protein leaflets, including the self-replication system, communication of living cells, and complex metabolic pathways.

### Limitations of study

A size recovery and re-fission of the asymmetric vesicles after fission is difficult because the amount of the residual oleosin in the asymmetric vesicles is not sufficient. By synthesizing oleosin into the asymmetric vesicles integrated by a cell-free protein synthesis system, size recovery and fission of the asymmetric vesicles will be repeated many times. This repeatable system for the size recovery and fission will contribute to a molecular or function evolution into the vesicles.

## STAR★Methods

### Key resources table

**Table:** 

REAGENT or RESOURCE	SOURCE	IDENTIFIER
**Bacterial and virus strains**		

*E. coli* strain BL21 (DE3)	Nippon Gene	Cat# 312-06534

**Chemicals, peptides, and recombinant proteins**		

1,2-dioleoyl-*sn*-glycero-3-phosphocholine (DOPC)	Avanti Polar Lipids	Cat# 850375
1-palmitoyl-2-hydroxy-*sn*-glycero-3-phosphocholine (16:0 lyso-PC)	Avanti Polar Lipids	Cat# 855675
1-(dipyrrometheneboron difluoride) undecanoyl-2-hydroxy-*sn*-glycero-3-phosphocholine (TopFluor lyso PC)	Avanti Polar Lipids	Cat# 810284
1,2-dioleoyl-*sn*-glycero-3-phospho-L-serine-N-(7-nitro-2-1,3-benzoxadiazol-4-yl) (NBD-DOPS)	Avanti Polar Lipids	Cat# 810198
1,2-dioleoyl-*sn*-glycero-3-phosphoethanolamine-N-(lissamine rhodamine B sulfonyl) (ammonium salt) (Rh-DOPE)	Avanti Polar Lipids	Cat# 810150
HEPES	Doujindo Molecular Technologies	Cat# 346-08235
Sodium Carbonate (Na2CO3)	Wako Pure Chemical Industries	Cat# 199-01585
Cobalt(Ⅱ) Chloride Hexahydrate	Wako Pure Chemical Industries	Cat# 036-03682
2,4,6-trinitrobenzenesulfonic acid sodium	Wako Pure Chemical Industries	Cat# 8209-10483
Oriole fluorescent gel stain	Bio-Rad	Cat# 1610496
Mineral oil	Merck KGaA	Cat# M3516-1L
Bacterial Protein Extraction Reagent (B-PER)	Thermo Fisher Scientific	Cat# 78248
Carboxyfluorescein	Tokyo Chemical Industry	Cat# C2477
Resorufin	Tokyo Chemical Industry	Cat# R0012
Alexa Fluor 546 NHS Ester (Succinimidyl Ester)	Thermo Fisher Scientific	Cat# A20002
Alexa Fluor 488 NHS Ester (Succinimidyl Ester)	Thermo Fisher Scientific	Cat# A20100

**Critical commercial assays**		

S30 T7 High-Yield Protein Expression System	Promega Corp	Cat# L1115

Recombinant DNA		

pET28a/oleosin	This paper	N/A
pET28a/OmpG	Tosaka et al.^54^	N/A
Amino acid sequence of oleosin MGLNDIFEAQKIEWHEGSLTHPQRQQQGPS TGKITGILFGLTGITLVGTVIGLALATPLFVIFSP VIVPAMIAIGLAVTGFLTSGTFGLTRSTMSVPV QRDYVKGKLQDVGEYLEHHHHHH	Vargo et al.^27^	N/A

**Software and algorithms**		

ImageJ	Schneider et al.^55^	https://imagej.net/ij/index.html

### Resource availability

#### Lead contact

Further information and requests for resources and reagents should be directed and will be fulfilled by the lead contact, Koki Kamiya (kamiya@gunma-u.ac.jp).

#### Materials availability

This work did not generate new unique reagents.

### Method details

#### Oleosin protein expression

Oleosin gene was incorporated into the vector pET28a using standard molecular biology techniques. The *Escherichia coli* strain BL21 (DE3) (Nippon Gene, Tokyo, Japan) was used to express oleosin protein. Cultures were grown at 37°C in Luria Bertani broth with kanamycin at a final concentration of 20 μg/mL. The oleosin cultures were diluted to OD_600_ ≈ 0.4, and expression was induced by isopropylthio-β-galactoside (IPTG) at a final concentration of 0.5 mM at 37°C for 4.5–9 h. The cultures were then centrifuged at 3,000 × *g* for 20 min, and the supernatant was discarded. The cell pellets were stored at −80°C.

#### Oleosin protein extraction and purification

A cell pellet collected from 50-mL cultures was resuspended in 4 mL B-PER containing 4 mg/mL lysozyme and 1 μg/mL DNase I and incubated at room temperature for 1 h. Then, 5 mL of B-PER diluted 1:10 (B-PER/water [v/v]) was added to the incubation solution and vortexed. The solution was centrifuged at 15,000 × *g* for 15 min, and the inclusion bodies were collected. The inclusion bodies were washed by vortexing for 1 min with 5 mL B-PER diluted 1:10 (B-PER/water [v/v]). The solution was centrifuged at 22,260 × *g* for 5 min, and the inclusion bodies were collected. The inclusion bodies were washed twice with 5 mL B-PER diluted 1:10 (B-PER/water [v/v]). The inclusion bodies were washed with 2 mL of 200 mM Na_2_CO_3_ (pH 11) by vortexing for 1 min. The solution was centrifuged at 22,260 × *g* for 5 min, and the inclusion bodies were collected. The inclusion bodies were washed twice with 2 mL of 200 mM Na_2_CO_3_ (pH 11). The resulting pellets were resuspended in 1 mL of 200 mM Na_2_CO_3_ (pH 11), and 7 mL of methanol and 2 mL of chloroform were added. The solution was centrifuged at 22,260 × *g* for 30 min, and the supernatant was collected. The supernatant was completely dried in a desiccator.

#### SDS-PAGE

The dried oleosin protein was dissolved in sucrose solution (10 mM HEPES, 140 mM NaCl, and 500 mM sucrose, pH 7.2), and the protein concentration was measured using a Nanodrop One (Thermo Scientific) (coefficients of molar absorbance at 280 nm: 8,480 M^−1^ cm^−1^). The oleosin proteins were separated using 15 w/v% SDS–PAGE. After electrophoresis, the gel was stained with Oriole Fluorescent Gel Stain and imaged with a LuminoGraph I (ATTO Corporation, Tokyo, Japan).

#### Oleosin emulsion preparation

The dried oleosin protein was dissolved in 8 M urea and 50 mM phosphate (pH 7.2), and the protein concentration was measured using a Nanodrop One. Alexa Fluor 488 carboxylic acid succinimidyl ester was conjugated to oleosin in the solution diluted by 10 mM HEPES, 140 mM NaCl (pH 7.2). Alexa Fluor 488 (15 μL, 1 mg/mL) was added to a 25 μL of solution containing 84 μg of oleosin. After incubation for 2 h at room temperature, using an Amicon Ultra centrifugal filter (MWCO 3 kDa), the non-conjugated Alexa Fluor 488 was completely removed from the solution using a sucrose solution containing 8 M urea and 50 mM phosphate. Then, 2.8 μL of 0.14–3.36 μg oleosin solution containing 5.6 mol % Alexa Fluor 488-conjugated oleosin (total final concentration 0.05–1.2 mg/mL) was added to 60 μL of mineral oil in a test tube and vortexed to generate the oleosin emulsions (Se-04, TAITEC, Saitama, Japan). The oleosin emulsions were observed using a CLSM with a 60× oil-immersion lens using a diode laser (473 nm) for Alexa Fluor 488 (485–554 nm).

#### Preparation lipid solution

DOPC, Rh-DOPE, and NBD-DOPS dissolved in chloroform were placed in a glass test tube. The solution was evaporated in a desiccator for 60 min to form a lipid film. The lipid film was dissolved in mineral oil (1.1 mM final DOPC concentration) by sonication over 100 min at 50°C. After sonication, the lipid solution was incubated overnight at room temperature.

#### Lipid–oleosin vesicle preparation

A DOPC monolayer containing 0.018**–**0.0018 mol % Rh-DOPE was prepared by pouring 30 μL of the DOPC solution on top of 30 μL of glucose solution (10 mM HEPES, 140 mM NaCl, and 500 mM glucose, pH 7.2) in a tube. The DOPC monolayer at the solution interface was assembled at 4°C for at least 5 h. To generate asymmetric vesicles containing DOPC on the outer leaflet and oleosin on the inner leaflet, an emulsion consisting of 2.8 μL of sucrose solution (with 3.36 μg oleosin) and 60 μL of mineral oil was added to the oil phase on the lipid monolayer. The tube was centrifuged at 5,200 × *g* for 10 min at room temperature in a desktop centrifuge (Chibitan-II Microcentrifuge 1P, Merck) to transfer the emulsions from the oil phase to the water phase. After centrifugation, the oil layer was removed from the tube, and asymmetric vesicles were collected at the bottom of the tube as clear aqueous solution. The lipid–oleosin vesicles were observed using an FV-1200 CLSM with a 60× oil-immersion lens using a diode laser (559 nm) for rhodamine (570–670 nm). The lipid–oleosin vesicles were prepared by centrifugation at four different speeds (100, 5,000, 10,000, and 15,000 × *g*) using a CF15RN centrifuge (Himac, Tokyo, Japan). After centrifugation, 20 μL of the lipid–oleosin vesicle solution was collected, diluted 4-fold with glucose solution (10 mM HEPES, 140 mM NaCl, and 500 mM glucose, pH 7.2), and observed using a CLSM. The experiment was performed three times for each parameter. The vesicle diameter was measured from rhodamine fluorescence on the vesicles using a line plot of ImageJ (NIH, Bethesda, MD, USA).

#### Lipid vesicles preparation by gentle hydration method

A 100 μL phospholipid solution of 1 mM DOPC and 1 mM DOPG (19:1 M ratio) dissolved in chloroform was added to a glass test tube (95 μL and 5 μL, respectively). The solution was evaporated under flowing argon gas, and lipid films were formed at the bottom of the tube. The films were completely dried in a desiccator and hydrated by adding 100 μL of sucrose solution (10 mM HEPES, 140 mM NaCl, and 500 mM sucrose, pH 7.2). The solution was incubated for more than 12 h at 27°C and then added to 500 μL of glucose solution.

#### Examination of the lipid asymmetry of lipid–oleosin vesicles

The lipid asymmetry of lipid–oleosin vesicles was confirmed by fluorescence quenching assay using Cobalt ion as an NBD quencher and asymmetric vesicles in the lipid monolayer of the outer leaflet containing 2 mol % NBD-DOPS. A total of 20 μL of the vesicle solution was collected from the tube. After observation, 5 μL of 50 mM NBD quencher dissolved in the glucose solution was added to the vesicle solution (final concentration of 10 mM). Time-lapse images of the NBD-quenched asymmetric vesicles were acquired using a CLSM. The NBD fluorescence intensity on the vesicles was measured using ImageJ.

#### Examination of the oleosin asymmetry of lipid-oleosin vesicles

To add of trypan blue into the outer solution, trypan blue was added to the outer solution of the lipid-oleosin vesicles containing the Alexa Fluor 546-conjugated oleosin at final concentration of 20 μg/mL, and the vesicles solution was observed using a CLSM. To add of trypan blue into the inner solution, the oleosin emulsions containing the Alexa Fluor 546-conjugated oleosin and trypan blue at final concentration of 20 μg/mL were transferred to the interface between the lipid and buffer solution to generate vesicles. The lipid-oleosin vesicles were observed using a CLSM. The Alexa Fluor 546-conjugated oleosin fluorescence intensities on the vesicles were measured using ImageJ.

#### Fluorescence recovery after photo bleaching (FRAP)

The mobility of lipid leaflet and oleosin leaflet were measured by FRAP technique. The asymmetric lipid-oleosin vesicles containing 1 mol % NBD-DOPS or the Alexa Fluor 546-conjugated oleosin were observed using a diode laser (473 nm) at 1% intensity or diode laser (559 nm) at 5% intensity, respectively. The laser power was set at 100% intensity during photobleaching. After photobleaching, the time-lapse CLSM images were acquired using CLSM.

#### Osmotic shock to the lipid-oleosin vesicles

Oleosin emulsions consisting of 2.8 μL 500 mM sucrose solution containing 3.92 μg oleosin and 60 μL mineral oil was added to the DOPC monolayer and centrifuged. The glucose solution was added to the outer solution of the lipid-oleosin vesicles at final glucose concentration to 500 mM, 1.5 M, and 2 M. After incubation at room temperature for 10 min, the vesicles were observed using a CLSM. The vesice diameter was measured from rhodamine fluorescnece on the vesicles using a line plot of ImageJ.

#### Cell-free synthesis of eGFP in asymmetric lipid–oleosin vesicles

eGFP in the asymmetric lipid–oleosin vesicles was synthesized using the S30 T7 High-Yield Protein Expression System. The cell-free synthesis solution was prepared according to the manufacturer’s protocol. Four microliters of S30 Premix Plus, 3.6 μL of T7 S30 Extract Circular, 2 μg of eGFP DNA, and 5 μg of oleosin protein were added to the tube and scaled up to 10 μL with sucrose solution. Then, 2.8 μL of the oleosin cell-free solution was added to 60 μL of mineral oil in a test tube and vortexed. Next, the generated emulsions were added to the DOPC monolayer. The tube was centrifuged at 5,000 × *g* for 10 min, and the vesicles were collected at the bottom of the tube. The vesicles were incubated at 30°C for 2.5 h and observed by a CLSM.

#### Insertion of Alexa Fluor 546-conjugated OmpG into lipid–oleosin vesicles

To insert OmpG into the outer solution of the lipid–oleosin vesicles, 1 μL of 538 nM Alexa Fluor 546-conjugated OmpG (final concentration: 26.9 nM) in a glucose buffer containing 0.0278% n-octyl-β-D-glucoside (OG) was added to 19 μL of the outer solution. The lipid–oleosin vesicles were incubated at 37°C for 30 min and observed using a CLSM. To insert OmpG into the inner solution of the lipid–oleosin vesicles, 0.7 μL of 108 nM Alexa Fluor 546-conjugated OmpG (final concentration: 26.9 nM) in sucrose buffer containing 0.00556% OG was added to 2.1 μL of 3.36 μg oleosin in sucrose solution. Oleosin emulsions were generated by vortexing a mixture of sucrose solution and mineral oil. Oleosin emulsions containing Alexa Fluor 546-conjugated OmpG were transferred to the interface between the lipid and buffer solution to generate vesicles. The lipid–oleosin vesicles were incubated at 37°C for 30 min and observed using a CLSM. As a negative control, 1 μL of 538 nM Alexa Fluor 546-conjugated OmpG (final concentration: 26.9 nM) in glucose buffer containing 0.0278% OG was added to 19 μL of the outer solution of lipid vesicles. The lipid vesicles were incubated at 37°C for 30 min and observed using a CLSM. The fluorescence of Alexa Fluor 546 on the vesicles was measured using ImageJ.

#### Observation of permeability via OmpG on lipid–oleosin vesicles

Lipid–oleosin vesicles containing 5 μM carboxyfluorescein were prepared using the droplet transfer method. In total, 20 μL of 0.85 μM OmpG containing 0.025% OG was added to 10 μL of lipid–oleosin vesicle solution. Time-lapse images were acquired using a CLSM. The fluorescence of carboxyfluorescein in these vesicles was measured using ImageJ.

#### Deformation and fission of lipid–oleosin vesicles

Oleosin emulsions consisting of 2.8 μL sucrose solution (with 3.92 μg or 0.28 μg oleosin) containing 100 μM resorufin and 60 μL mineral oil was added to the DOPC monolayer and centrifuged at 5,200 × *g* for 10 min at room temperature. Ten microliters of vesicle solution were collected from the tube. Ten microliters of 500 μM or 30 μM 16:0 lyso-PC micelles containing 0.14 mol % TopFluor lyso-PC was added to 10 μL of the lipid–oleosin vesicle solution. Time-lapse images were acquired using a CLSM. The TopFluor lyso-PC fluorescence and resorufin fluorescence were observed using a CLSM with a 60× oil-immersion lens using a diode laser (473 nm for TopFluor) and (559 nm for resorufin), respectively. The area of the vesicle was measured using a feel hand tool of ImageJ. The fluorescence of TopFluor on these vesicles was measured using ImageJ.

### Quantification and statistical analysis

Statistical analyses were performed using Origin (version 9.7.5.184) software. The value of n represencts the number of vesicles. The results were reported as mean ± SD and analyzed by *t* tset. p values were considered statistically significant.

## Data Availability

• All data reported in this paper will be shared by the lead contact upon request. • This paper does not report original code. • Any additional information required to reanalyze the data reported in this paper is available from the lead contact upon.
