# Supplementary material for: Cell-sized asymmetric phospholipid-amphiphilic protein vesicles with growth, fission, and molecule transportation
Source: iScience. 2023 Jan 31;26(3):106086. doi: 10.1016/j.isci.2023.106086 (PMC9950948; doi:10.1016/j.isci.2023.106086)
Supplement: Document S1. Figures S1-S23 and Tables S1-S3 [file mmc1.pdf]

iScience, Volume *26*

Supplemental information

Cell-sized asymmetric phospholipid-amphiphilic protein vesicles with growth, fission, and molecule transportation

Masato Suzuki and Koki Kamiya

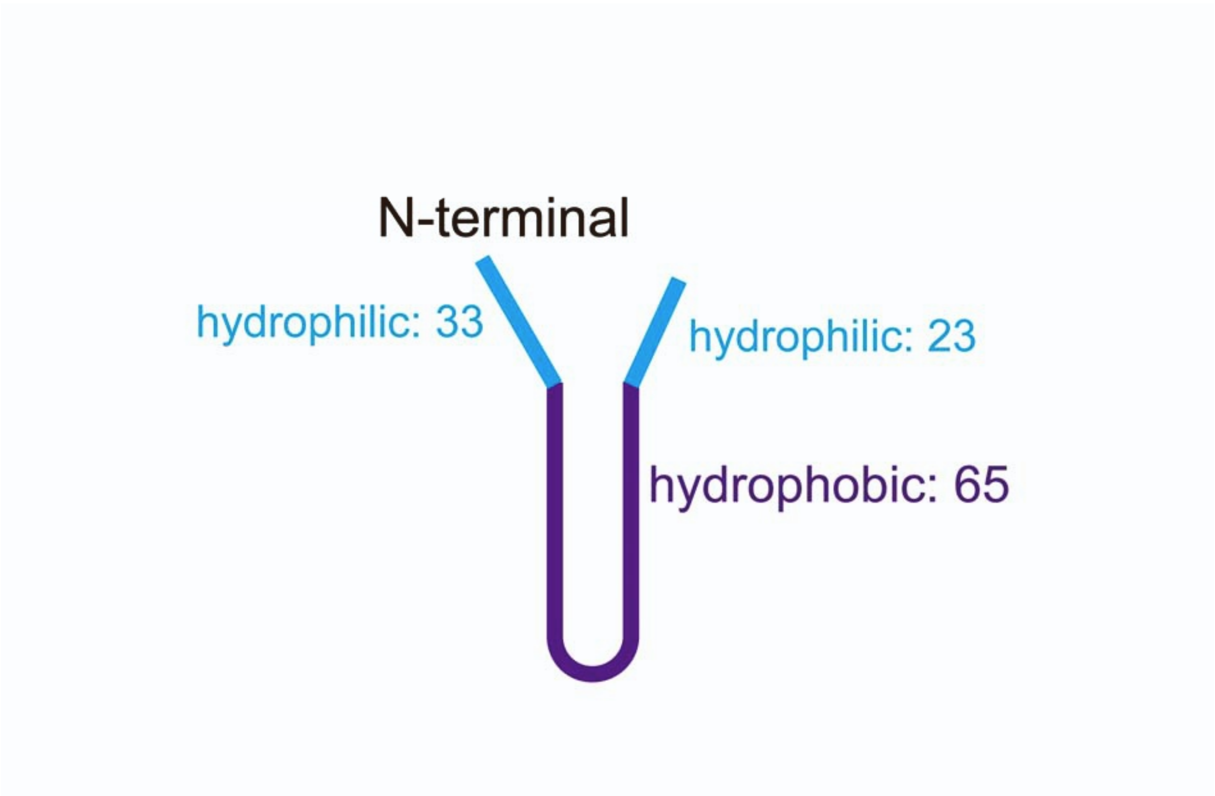

Figure S1. Schematic representation of oleosin molecular. The hydrophilic segments are shown in blue and the hydrophobic core is shown in purple. Numbers indicate the number of amino acids in each segment.

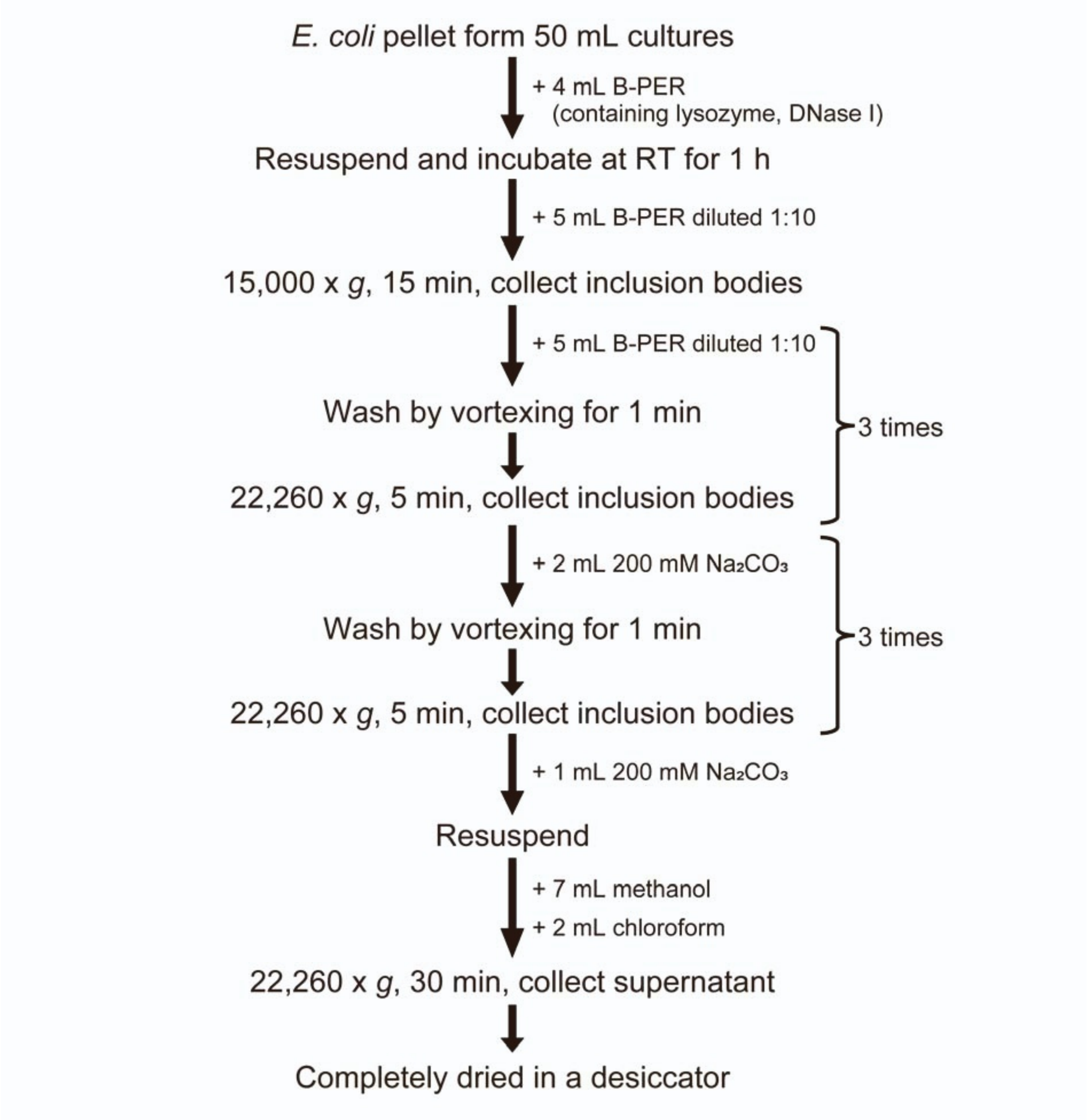

Figure S2. Flowchart of oleosin protein purification method, related to STAR Methods.

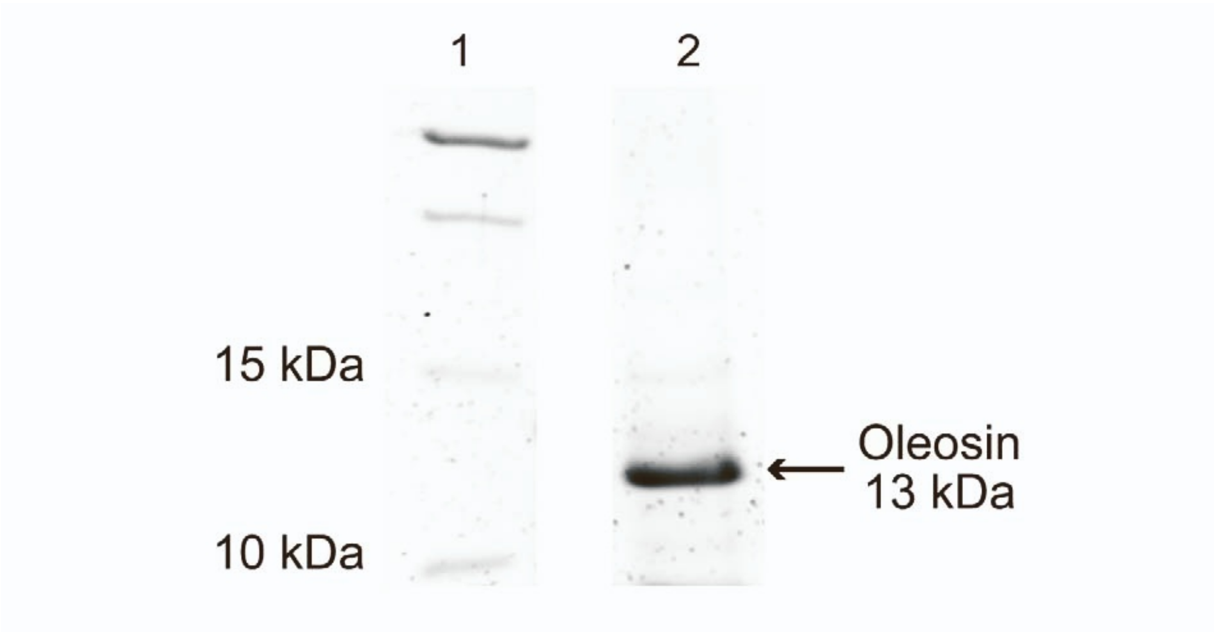

Figure S3. SDS-PAGE of purified oleosin, related to Figure 1. Lane1: standard protein ladder, lane2: purified oleosin protein.

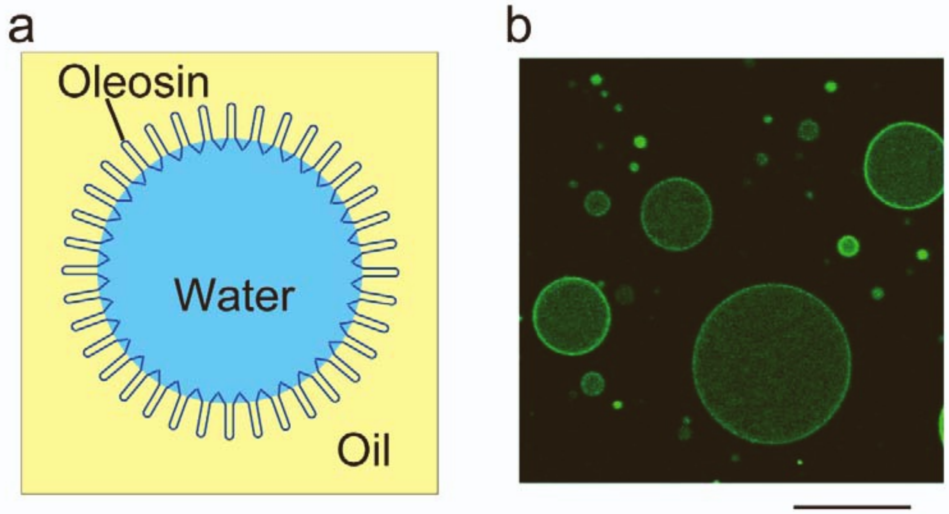

Figure S4. Water-in-oil (w/o) emulsion formed by Alexa Fluor 488-conjugated oleosin, related to Figure 1. (a) Schematic representation of w/o emulsion with Alexa Fluor 488-conjugated oleosin arranged between oil and water interface. (b) Typical confocal image of Alexa Fluor 488-conjugated oleosin emulsions. Scale bar, 20 μm.

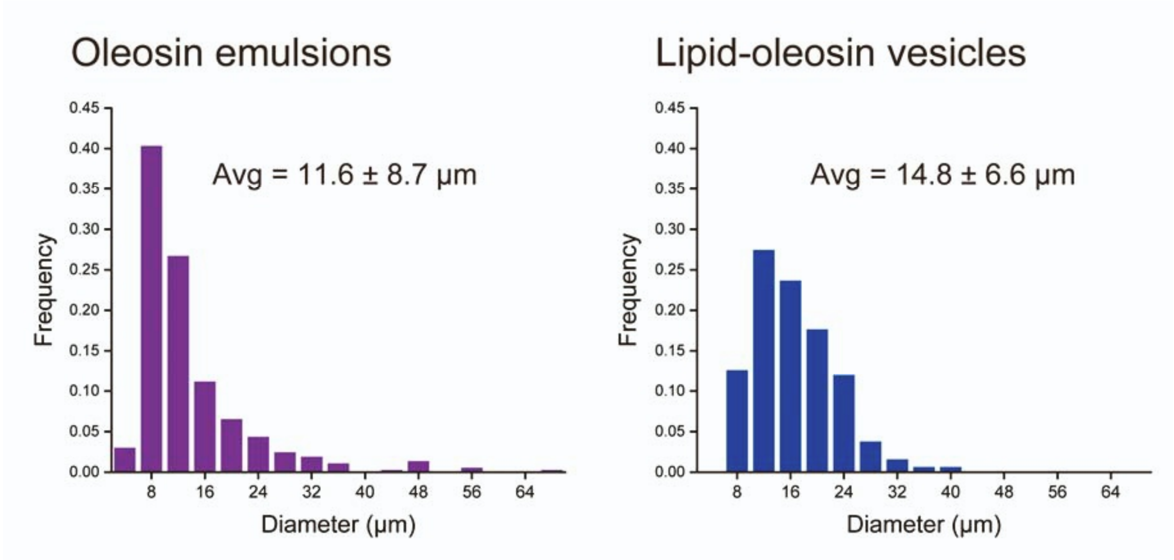

Figure S5. Size distribution of the oleosin emulsions (*n* = 367) and the lipid-oleosin vesicles (*n* = 320) prepared by centrifugal force applied at 5,200 × g, related to Figure 2.

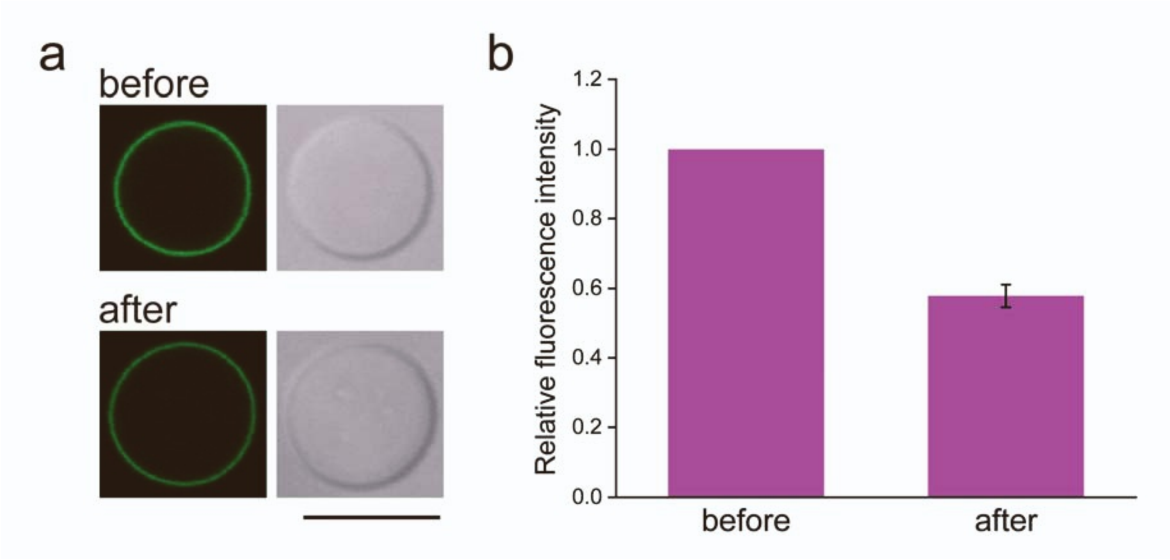

Figure S6. NBD quenching assay of the lipid vesicles, related to Figure 3. (a) Typical confocal images of the symmetric lipid vesicles containing NBD-DOPS before and 20 minutes after addition of the quencher. Scale bar, 10 μm. (b) Relative fluorescence intensities of NBD on the lipid vesicles before (n = 11) and after (n = 11) addition of quencher. Background fluorescence intensities were excluded. Data represented as mean ± SD.

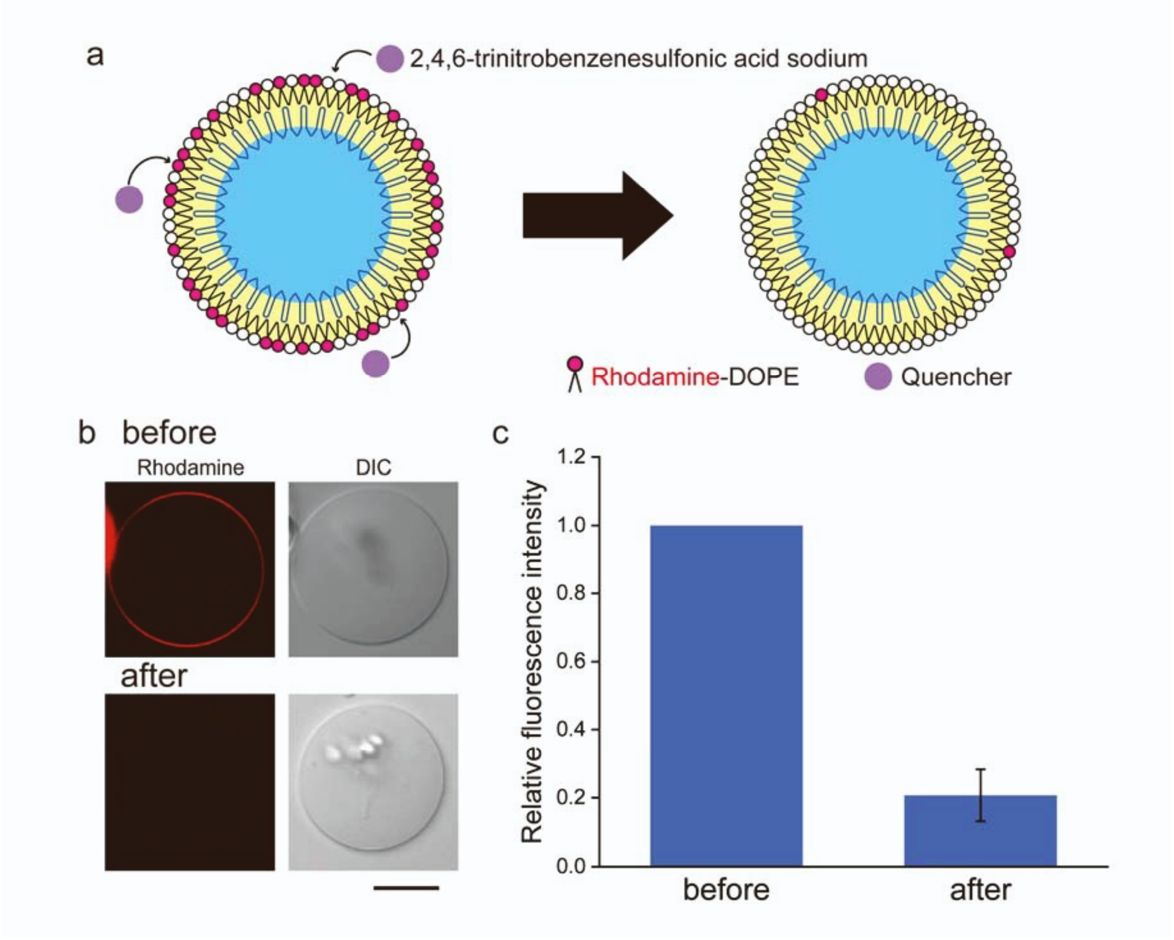

Figure S7. Rhodamine quenching assay to confirm the lipid asymmetricity of the lipid–oleosin vesicles, related to Figure 3. (a) Schematic representation of quenching assay using 2,4,6-trinitrobenzenesulfonic acid sodium (quencher). (b) Typical confocal images of the lipid–oleosin vesicles containing Rh-DOPE on the outer leaflet before and 1 to 10 minutes after addition of the quencher. Scale bar, 10 μm. (c) Fluorescence intensities of rhodamine on the lipid–oleosin vesicles before (n = 17) and after (n = 17) addition of quencher. Background fluorescence intensities were excluded. Data represented as mean ± SD.

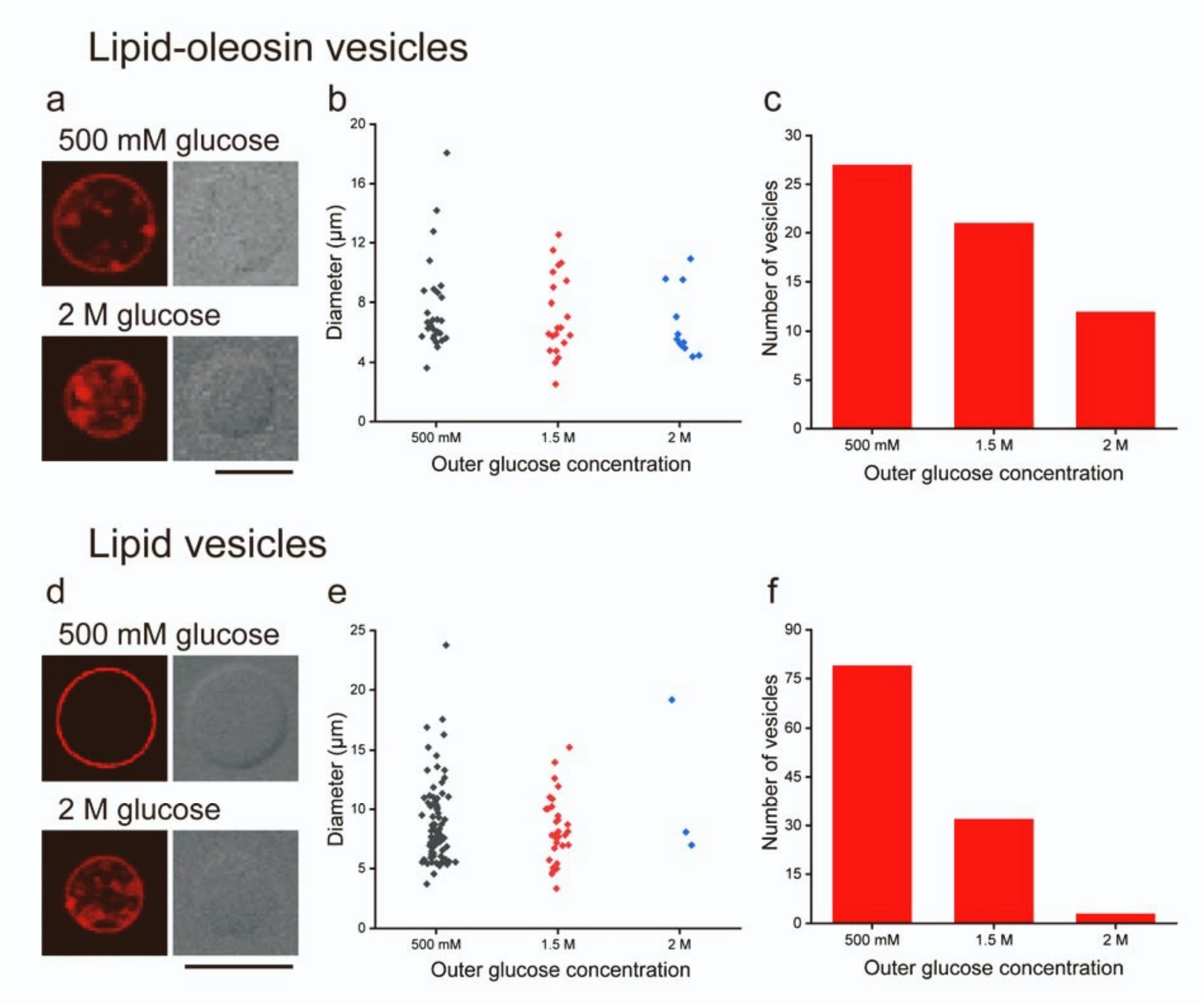

Figure S8. Invesigation of membrane stability of the asymmetric lipid-oleosin by changing the glucose solution in the outer solution, related to Figure 4. (a) Typical confocal images of the lipid-oleosin vesicles with the outer final glucose concentration of 500 mM and 2 M. Scale bar, 5 μm. (b), (c) Diameters and number of the lipid-oleosin vesicles after 10 min incubation at room temperature with different outer final glucose concentrations (*n* = 27 [500 mM], 21 [1.5 M], and 12 [2 M]). The number of processed images were 26 [500 mM], 20 [1.5 M], and 11 [2 M]. (d) Typical confocal images of the DOPC lipid vesicles (formed by droplet transfer method) with the outer final glucose concentration of 500 mM and 2 M. Scale bar, 10 μm. (e), (f) Diameters and number of the lipid vesicles after 10 min incubation at room temperature with different outer final glucose concentrations (*n* = 79 [500 mM], 32 [1.5 M], and 3 [2 M]). The number of processed images were 64 [500 mM], 27 [1.5 M], and 3 [2 M].

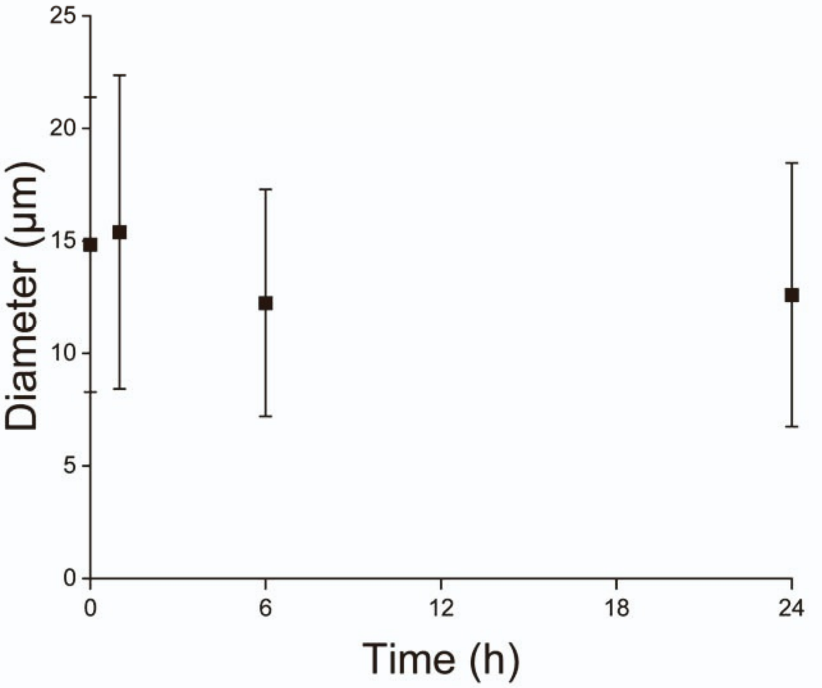

Figure S9. Observation of the size stability of the asymmetric lipid-oleosin vesicles, related to Figure 5. Average diameters of the asymmetric lipid-oleosin vesicles before (n = 320) and after incubation at 37 ℃ for 1 h (n = 214), 6 h (n = 398), and 24 h (n = 236) (new vesicles were observed at each time point). Data represented as mean ± SD.

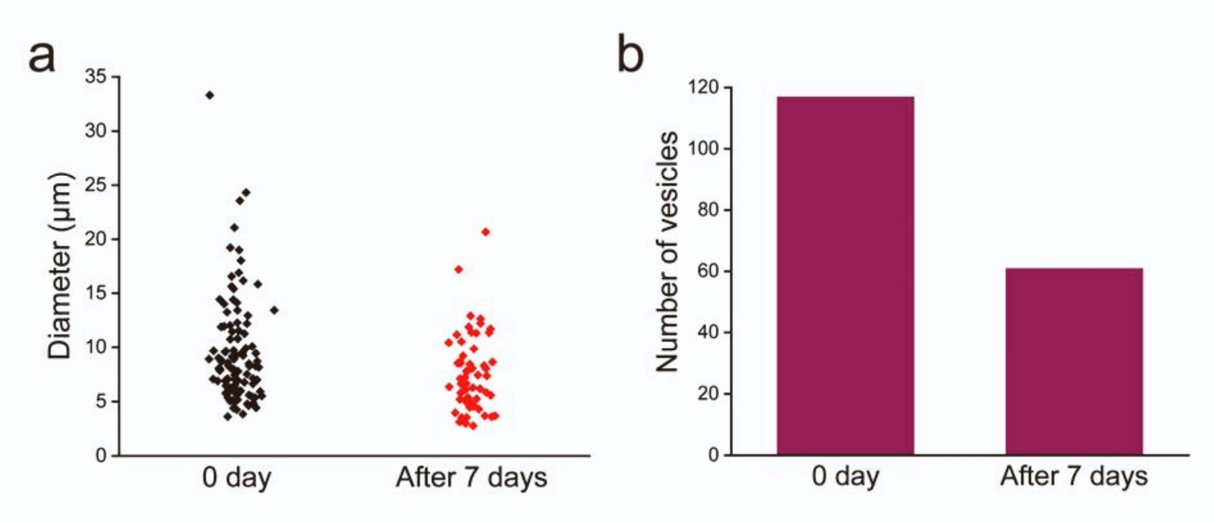

Figure S10. Long-term stability of the asymmetric lipid-oleosin vesicles, related to Figure 5. (a) Diameters of the asymmetric lipid-oleosin vesicles before (n = 117) and after (n = 61) incubation at 37 ℃ for 7 days. The number of processed images was 84 [before incubation] and 57 [after incubation]. (b) The number of asymmetric lipid-oleosin vesicles obtained before and after incubation at 37 ℃ for 7 days. The number of processed images was 84 [before incubation] and 57 [after incubation].

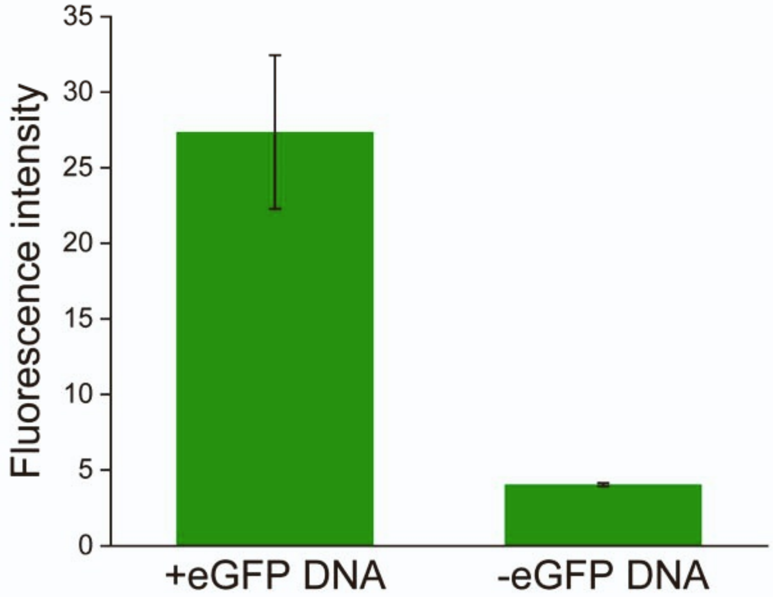

Figure S11. Fluorescence intensities of eGFP in the lipid-oleosin vesicles with (*n* = 5) or without (*n* = 10) eGFP-encoded DNA, related to Figure 5. Data represented as mean ± SD.

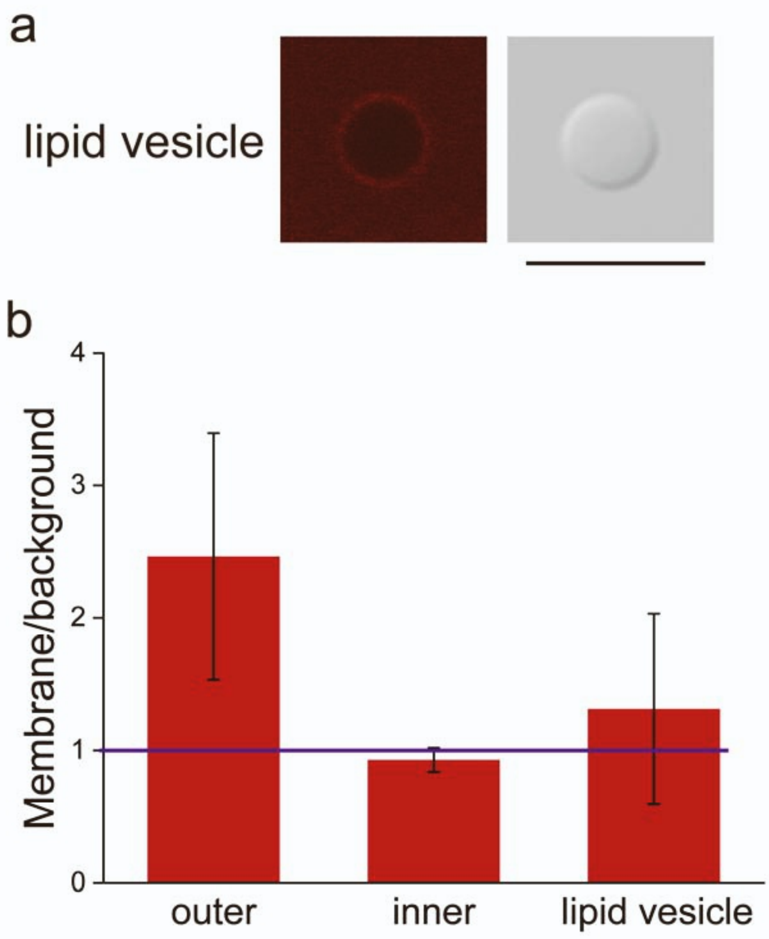

Figure S12. Insertion of OmpG into asymmetric lipid-oleosin vesicles and lipid vesicles, related to Figure 6. (a) Typical confocal images of the lipid vesicles with Alexa Fluor 546-conjugated OmpG from the outside. Scale bar, 10 μm. (b) Membrane fluorescence intensity of the lipid vesicles with Alexa Fluor 546-conjugated OmpG from the outside was normalized by the background fluorescence intensity (*n* = 51). Data represented as mean ± SD.

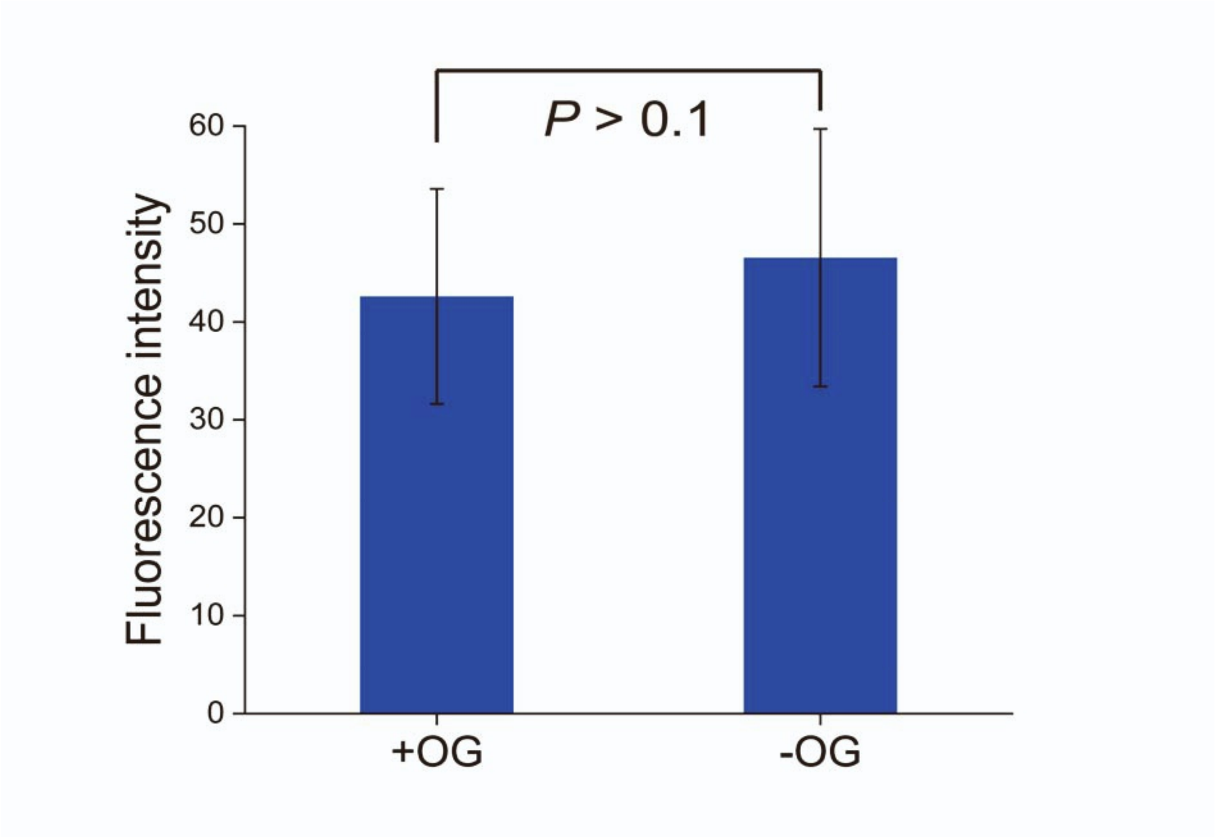

Figure S13. Fluorescence intensities of carboxyfluorescein in the lipid-oleosin vesicles with (*n* = 12) or without (*n* = 14) n-octyl-β-D-glucoside (OG) into the outer solution, related to Figure 6. Data represented as mean ± SD.

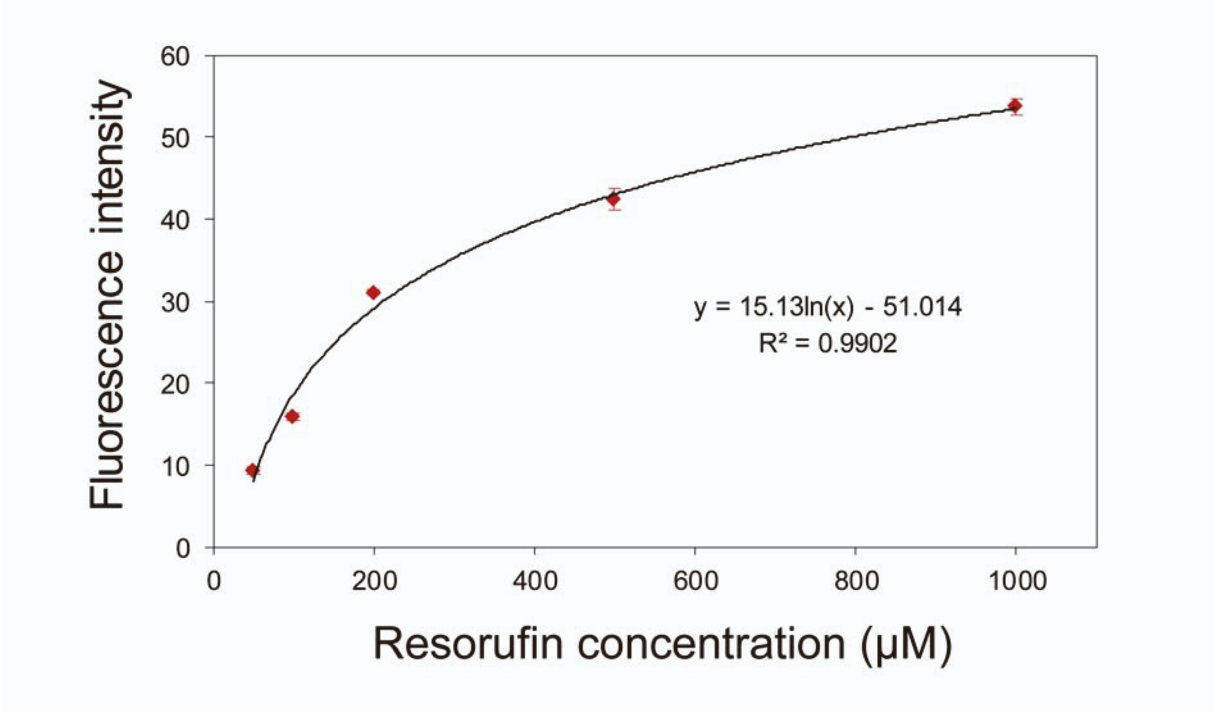

Figure S14. Calibration curve for conversion from fluorescence intensities to concentration of resorufin, related to Figure 7. Data represented as mean ± SD.

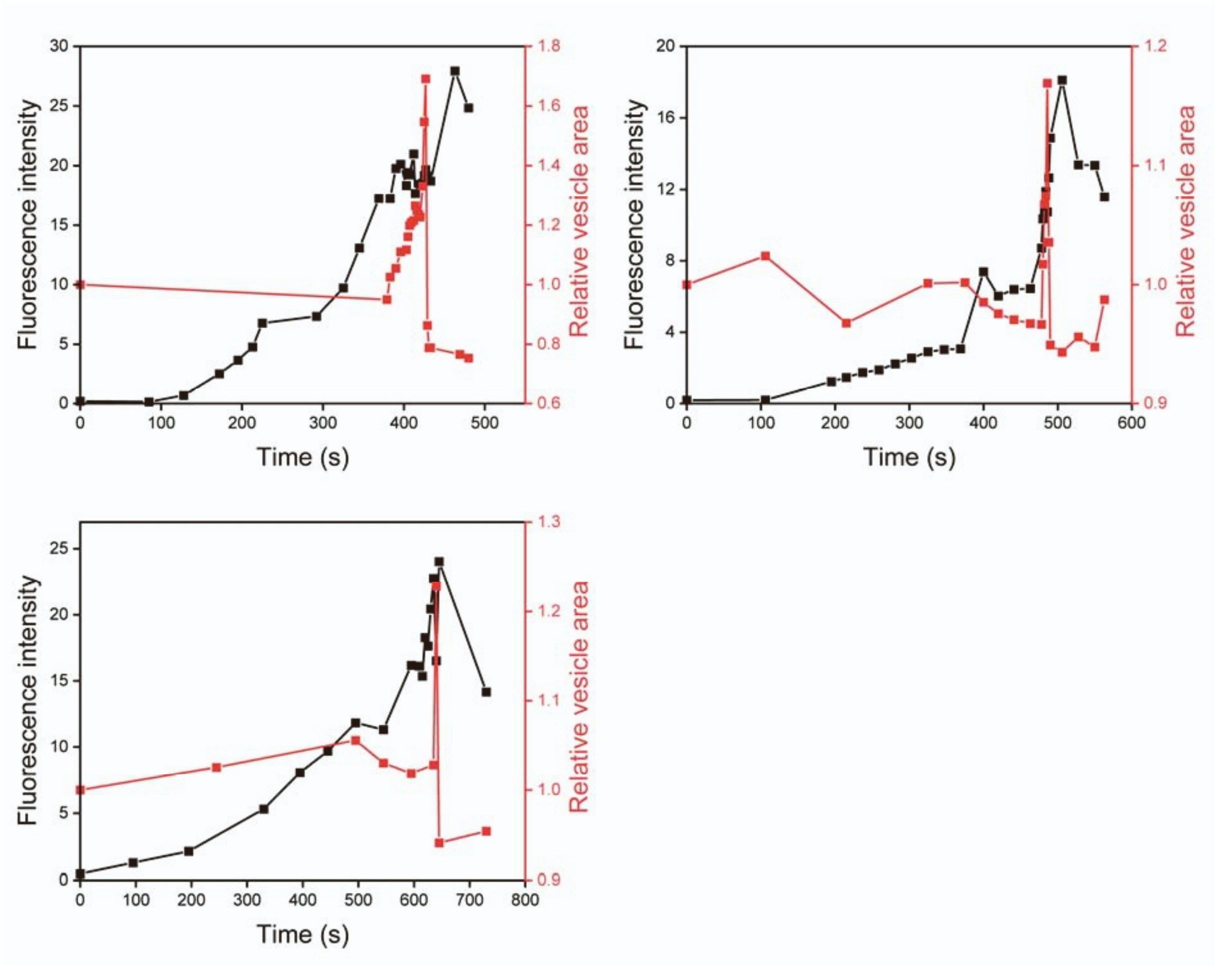

Figure S15. Fluorescence intensities of the TopFluor lyso-PC on the lipid-oleosin vesicle membranes and relative vesicle area of the lipid-oleosin vesicles after the addition of TopFluor lyso-PC micelles (final concentration: 250 μM) (3 experiments), related to Figure 7. Black lines show the fluorescence intensities of the TopFluor lyso-PC on the lipid-oleosin vesicle membranes after the addition of the TopFluor lyso-PC micelles. Red lines show the relative vesicle area of the lipid-oleosin vesicles after the addition of the TopFluor lyso-PC micelles.

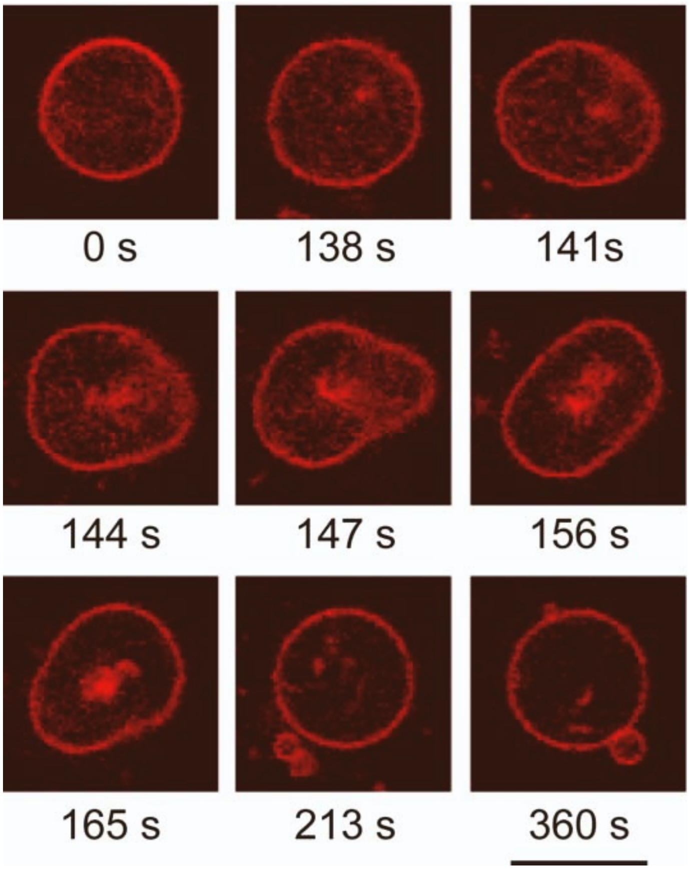

Figure S16. Deformation and fission of the lipid-oleosin vesicles containing lyso-PC, related to Figure 7. Typical CLSM time-lapse images of deformation and fission of the lipid-oleosin vesicle after the addition of lyso-PC. Scale bar, 10 μm.

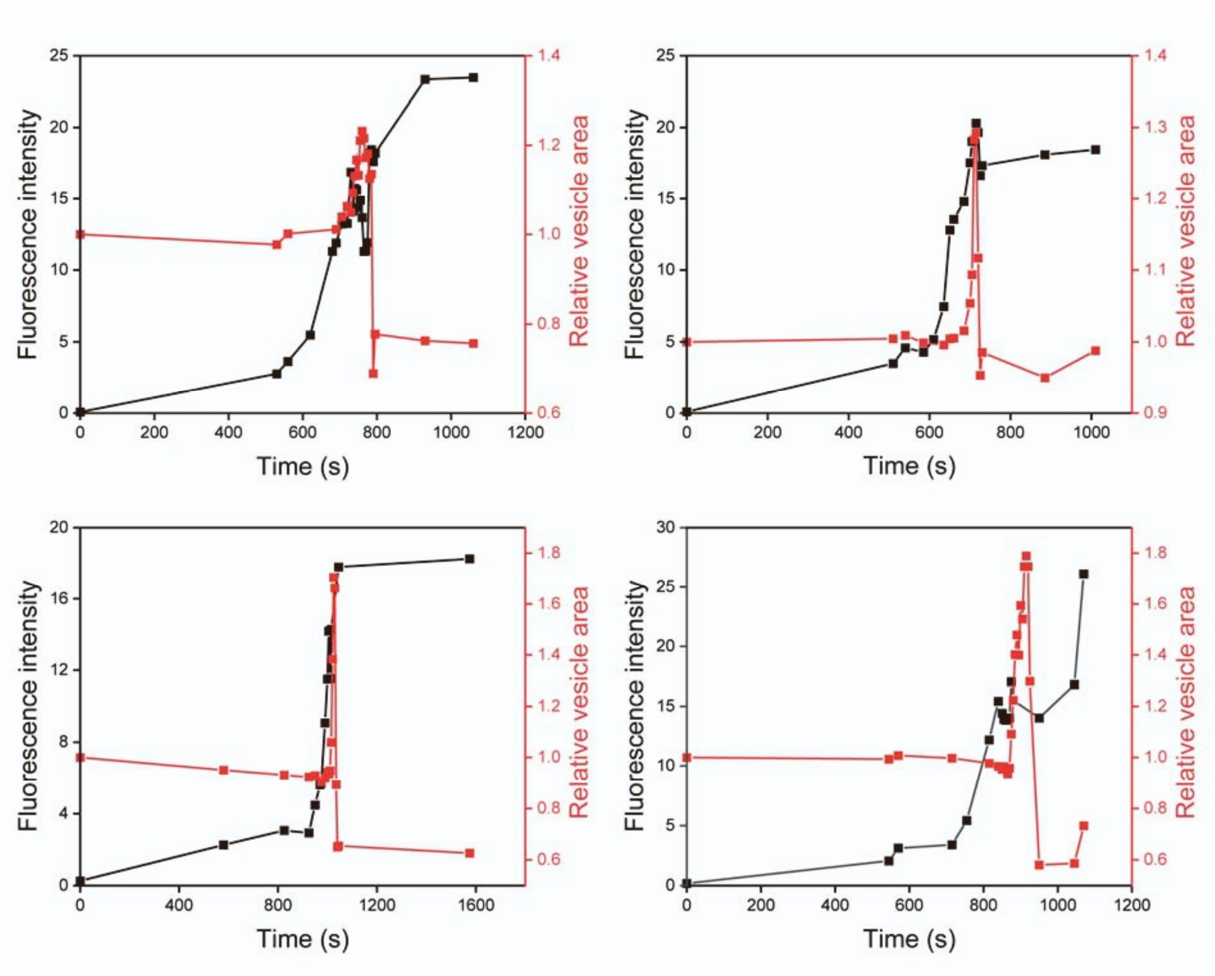

Figure S17. Fluorescence intensities of the TopFluor lyso-PC on the lipid-oleosin vesicle membranes and relative vesicle area of the lipid-oleosin vesicles after the addition of TopFluor lyso-PC micelles (final concentration: 15 μM) (4 experiments), related to Figure 7. Black lines show the fluorescence intensities of the TopFluor lyso-PC on the lipid-oleosin vesicle membranes after the addition of the TopFluor lyso-PC micelles. Red lines show the relative vesicle area of the lipid-oleosin vesicles after the addition of the TopFluor lyso-PC micelles.

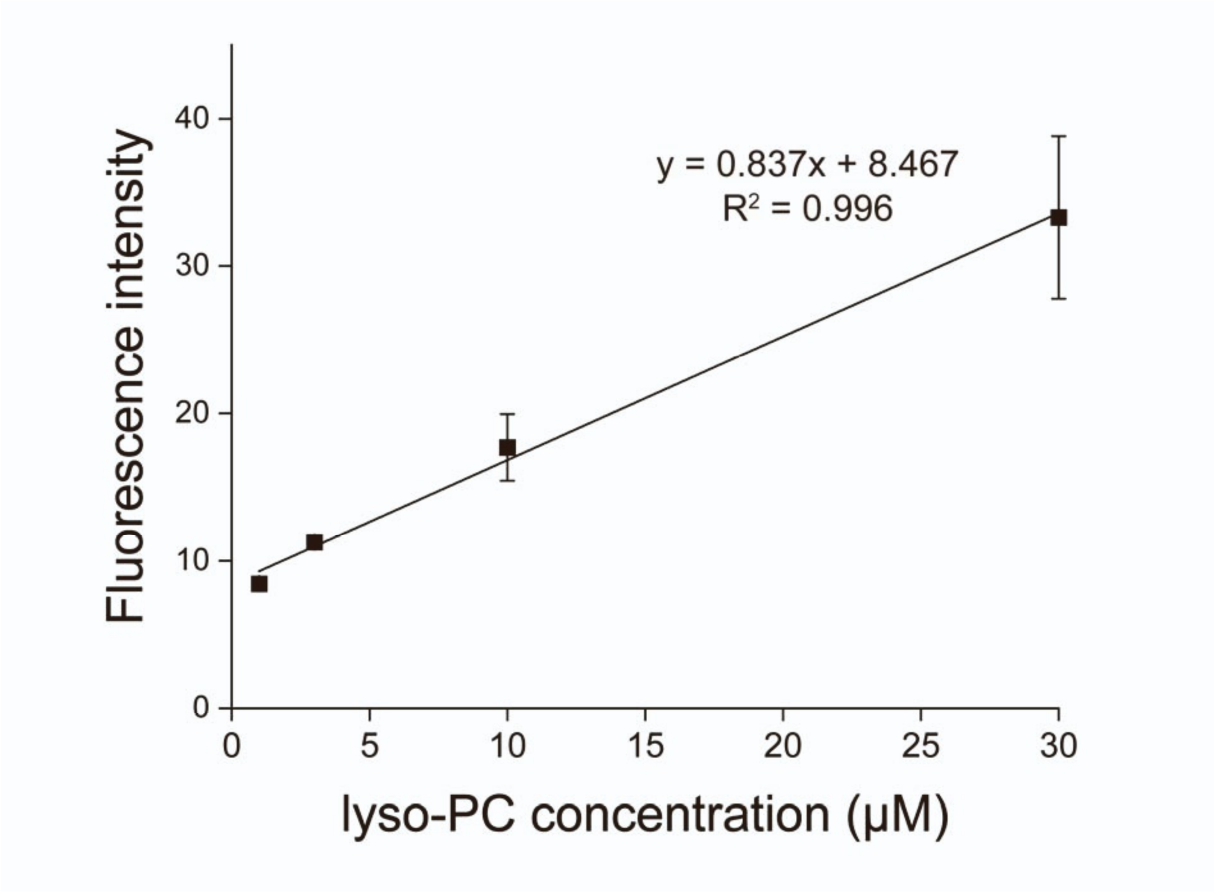

Figure S18. Calibration curve for conversion from the fluorescence intensity of fluorescence intensities of TopFluor lyso-PC to the concentration of lyso-PC, related to Figure 7. Error bar, standard deviation. Calibration curve for conversion from fluorescence intensities of TopFluor lyso-PC to concentration of lyso-PC: Ten microliters of 1 μM, 3 μM, 10 μM, and 30 μM 16:0 lyso-PC micelles containing 0.14 mol% TopFluor lyso-PC was observed by the CLSM. The fluorescence of TopFluor lyso-PC was measured using ImageJ. Data represented as mean ± SD.

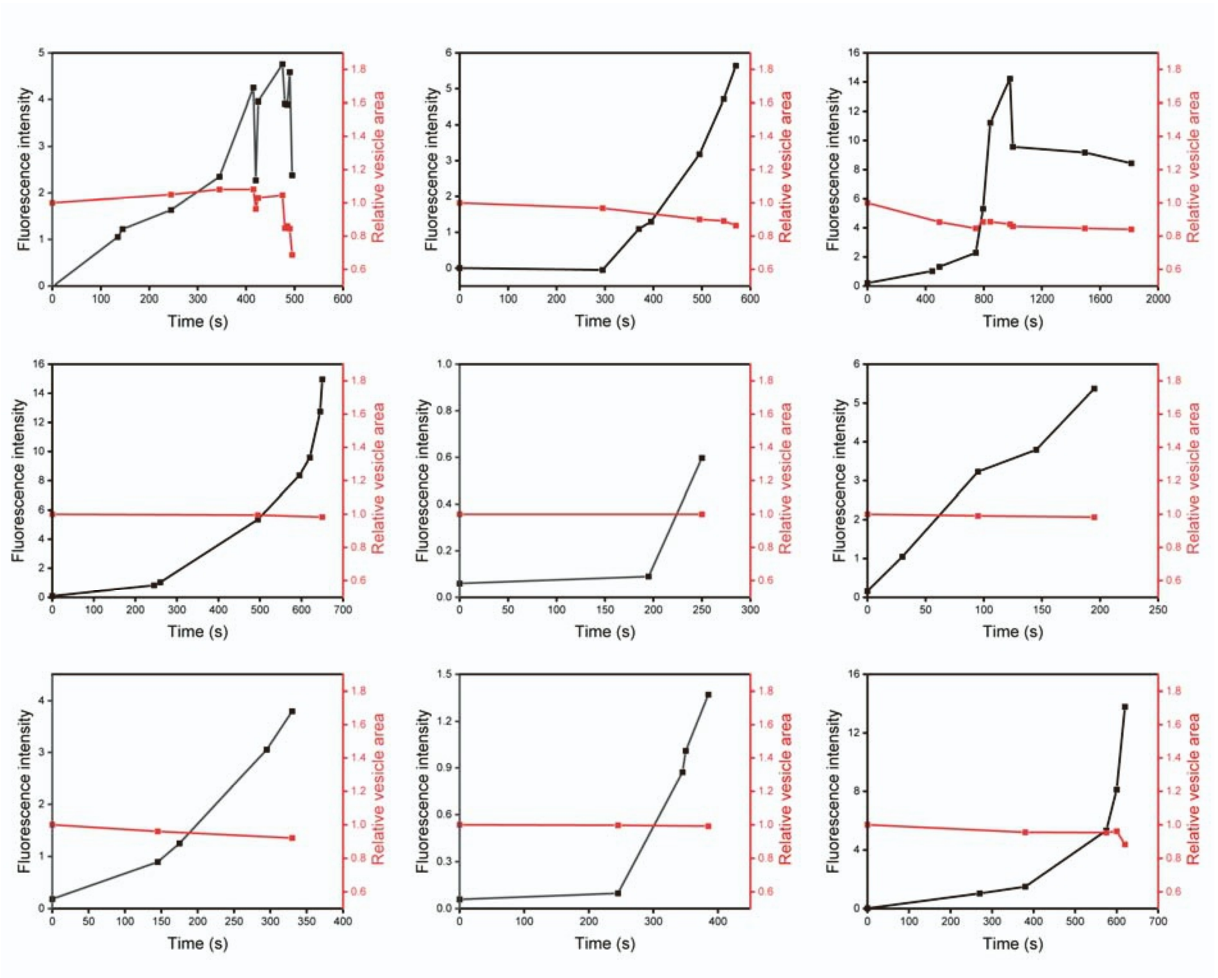

Figure S19. Relative vesicle area and fluorescence intensities of the TopFluor lyso-PC on the membrane lipid-oleosin vesicle containing 0.28 μg oleosin after the addition of TopFluor lyso-PC micelles (final concentration: 250 μM) (9 experiments), related to Figure 7. Black lines show the fluorescence intensities of the TopFluor lyso-PC on the lipid-oleosin vesicle membranes after the addition of the TopFluor lyso-PC micelles. Red lines show the relative vesicle area of the lipid-oleosin vesicles after the addition of the TopFluor lyso-PC micelles.

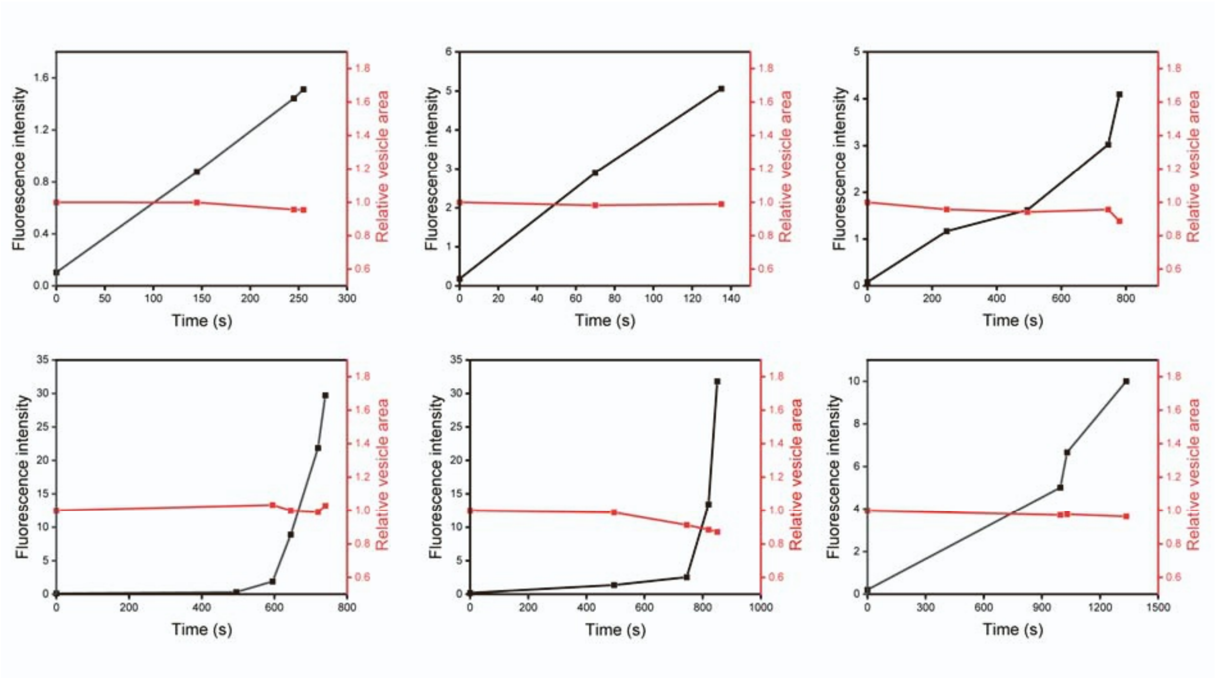

Figure S20. Fluorescence intensities of the TopFluor lyso-PC on the lipid vesicle membranes and relative vesicle area of the lipid vesicles after the addition of TopFluor lyso-PC micelles (final concentration: 250 μM) (6 experiments), related to Figure 7. Black lines show the fluorescence intensities of the TopFluor lyso-PC on the lipid-oleosin vesicle membranes after the addition of the TopFluor lyso-PC micelles. Red lines show the relative vesicle area of the lipid-oleosin vesicles after the addition of the TopFluor lyso-PC micelles.

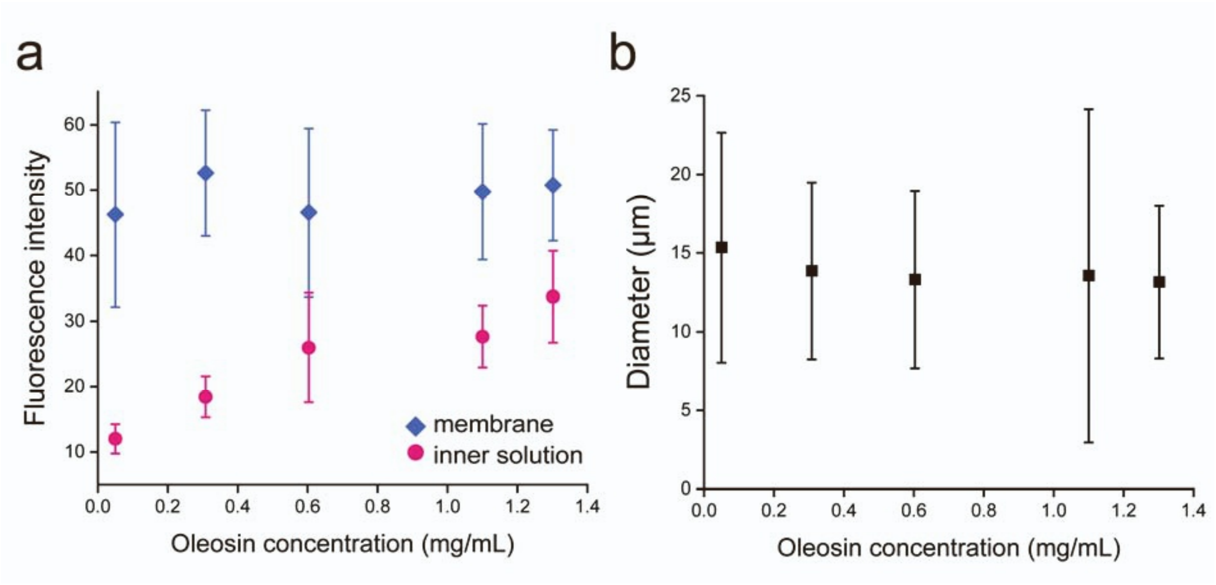

Figure S21. Fluorescence intensities of the membrane and inner solution of the oleosin emulsions and the diameters of the oleosin emulsions by changing the oleosin concentration, related to Figure 7. (a) Fluorescence intensities on the membranes and inner solution of the oleosin emulsions at each oleosin concentration (*n* = 22). Error bar, standard deviation. (b) Average diameters of the oleosin emulsions of each oleosin concentration (*n* = 22). Background fluorescence intensities were excluded. Data represented as mean ± SD.

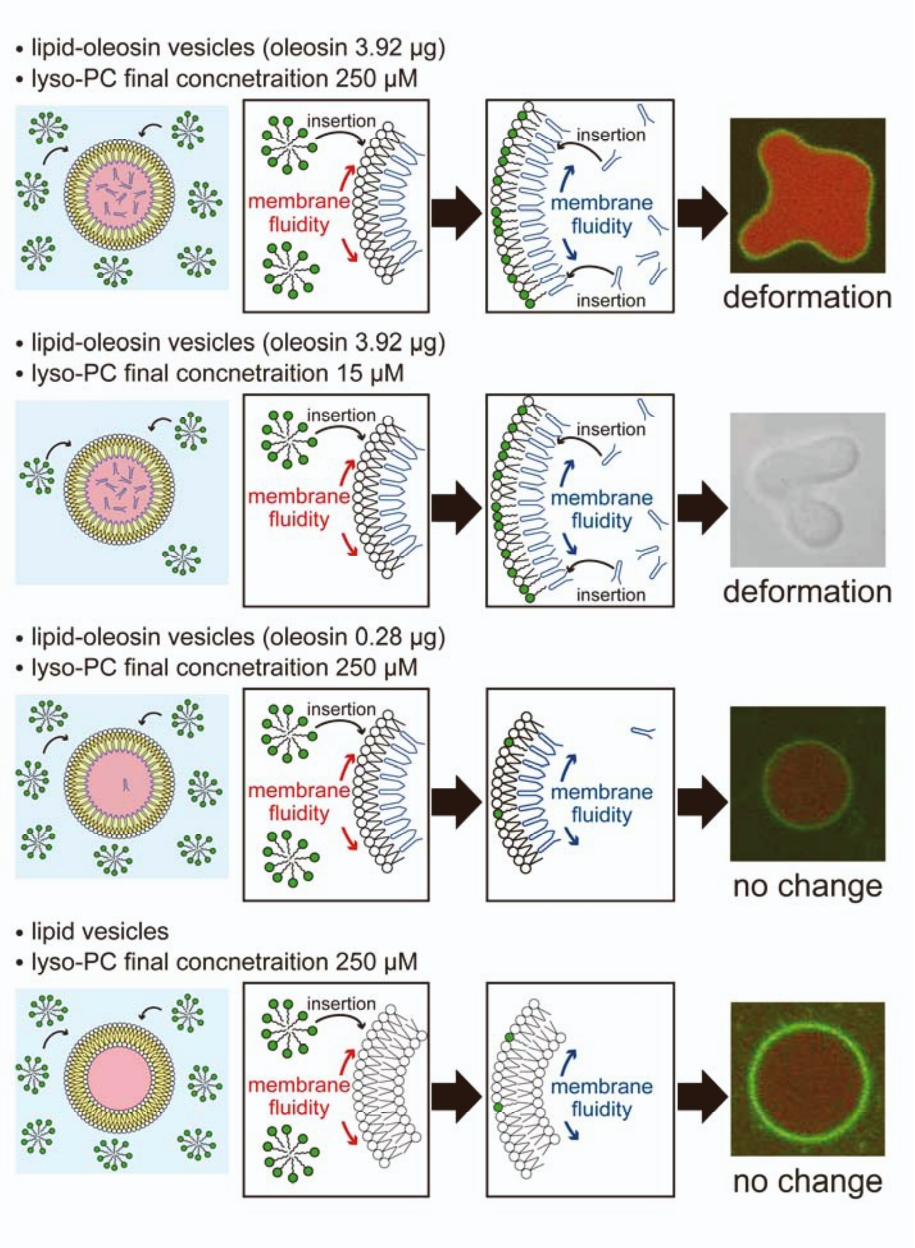

Figure S22. Shematic representation of membrane behavior of the lipid-olesin vesicles and lipid vesicles after the addition of lyso-PC micelles, related to Figure 7.

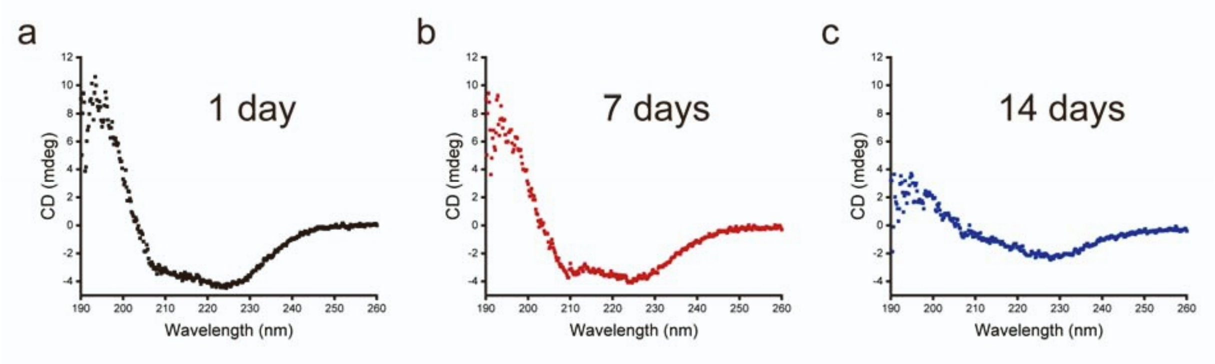

Figure S23. Evaluation of oleosin structure using circular dichroism (CD) spectroscopy, related to Figure 7. (a), (b), (c) CD spectrum of oleosin after purification for 1, 7, and 14 days, respectively.

Table S1. Resorufin concentration of inner solution and vesicle volume in spherical and deformation condition of the lipid-oleosin vesicles. Resorufin concentration was measured by resorufin fluorescence intensities in the lipid-oleosin vesicles. The lipid-oleosin vesicle volume in the deformation condition was estimated by the resorufin concentration in the spherical and deformation condition.

		resorufin concentration in the vesicle (μM)	vesicle volume (μm^3)
vesicle 1	spherical condition	487.69	241.71
	deformation conditon	480.02	245.58
vesicle 2	spherical condition	493.27	144.00
	deformation conditon	466.23	152.36
vesicle 3	spherical condition	295.59	218.14
	deformation conditon	239.62	269.10

Table S2. Each value of the deformation and fission of the lipid-oleosin vesicles.

final concentration of lyso-PC micelles	250 μM (n = 3)	15 μM (n = 4)
time of maximal relative vesicle area	425-640 (s)	715-1025 (s)
area of maximal relative vesicle	1.17-1.69	1.23-1.79
TopFluor fluorescence intensities of maximal relative vesicle area	10.7-19.7	13.6-20.3
area of relative fission vesicle	0.19-0.47	0.18-0.70
area of relative original vesicle after fission	0.79-0.99	0.59-0.99

Table S3. Details of each value of the deformation and fission of the lipid-oleosin vesicles.

final concentration of lyso-PC micelles	250 μM			15 μM			
time of maximal relative vesicle area	425 (s)	490 (s)	640 (s)	760 (s)	715 (s)	1025 (s)	915 (s)
area of maximal relative vesicle	1.69	1.17	1.23	1.23	1.29	1.7	1.79
TopFluor fluorescence intensities of maximal relative vesicle area	19.66	10.74	16.51	13.67	20.31		
area of relative fission vesicle	0.36	0.11	0.24	0.53	0.1	0.29	0.64
	0.1	0.08			0.04	0.19	0.26
					0.01	0.09	0.1
					0.01		
area of relative original vesicle after fission	0.79	0.99	0.95	0.69	0.99	0.65	0.59
